# Overexpression of a Sugarcane BAHD Acyltransferase Alters Hydroxycinnamate Content in Maize Cell Wall

**DOI:** 10.3389/fpls.2021.626168

**Published:** 2021-04-21

**Authors:** Amanda Fanelli, David M. Rancour, Michael Sullivan, Steven D. Karlen, John Ralph, Diego Mauricio Riaño-Pachón, Renato Vicentini, Tatiane da Franca Silva, André Ferraz, Ronald D. Hatfield, Elisson Romanel

**Affiliations:** ^1^Laboratório de Genômica de Plantas e Bioenergia (PGEMBL), Departamento de Biotecnologia, Escola de Engenharia de Lorena, Universidade de São Paulo, Lorena, Brazil; ^2^Lytic Solutions, LLC, Madison, WI, United States; ^3^U.S. Dairy Forage Research Center, Agricultural Research Service, U.S. Department of Agriculture, Madison, WI, United States; ^4^Department of Biochemistry, and The Department of Energy’s Great Lakes Bioenergy Research Center, The Wisconsin Energy Institute, University of Wisconsin, Madison, WI, United States; ^5^Laboratório de Biologia Computacional, Evolutiva e de Sistemas, Centro de Energia Nuclear na Agricultura, Universidade de São Paulo, Piracicaba, Brazil; ^6^Departamento de Genética e Evolução, Instituto de Biologia, Universidade Estadual de Campinas, Campinas, Brazil; ^7^Laboratório de Ciências da Madeira, Departamento de Biotecnologia, Escola de Engenharia de Lorena, Universidade de São Paulo, Lorena, Brazil

**Keywords:** BAHD acyltransferases, biomass engineering, biorefineries, ferulic acid (FA), *p*-coumaric acid (*p*CA)

## Abstract

The purification of hydroxycinnamic acids [*p*-coumaric acid (*p*CA) and ferulic acid (FA)] from grass cell walls requires high-cost processes. Feedstocks with increased levels of one hydroxycinnamate in preference to the other are therefore highly desirable. We identified and conducted expression analysis for nine BAHD acyltransferase *ScAts* genes from sugarcane. The high conservation of AT10 proteins, together with their similar gene expression patterns, supported a similar role in distinct grasses. Overexpression of *ScAT10* in maize resulted in up to 75% increase in total *p*CA content. Mild hydrolysis and derivatization followed by reductive cleavage (DFRC) analysis showed that *p*CA increase was restricted to the hemicellulosic portion of the cell wall. Furthermore, total FA content was reduced up to 88%, resulting in a 10-fold increase in the *p*CA/FA ratio. Thus, we functionally characterized a sugarcane gene involved in *p*CA content on hemicelluloses and generated a C4 plant that is promising for valorizing *p*CA production in biorefineries.

## Introduction

Grasses have great importance to worldwide agriculture, as huge volumes are used as food, animal feed, and bioenergy sources. The processing of grasses in agroindustry generates several non-grain products, such as stover or straw. The composition of these lignocellulosic materials makes them suitable feedstocks for the production of second-generation fuels and other value-added products, in a biorefinery concept (Chandel et al., 2012; Jin et al., 2018). Hydroxycinnamic acids, both ferulic acid (FA) and *p*-coumaric acid (*p*CA), are high-value chemicals that could be produced in this context. These phenolic acids present several health benefits due to their antioxidant, anti-inflammatory, and antimicrobial properties, being widely used in food, pharmaceutical, and cosmetics industries (Ou and Kwok, 2004; Pei et al., 2016). Currently, FA is often extracted from rice bran oil (20-25% wt of rice bran), which contains 0.9-2.9% of esters of *trans*-ferulic acid. *p*CA can be extracted from plants, such as *Hedyotis diffusa*, an herb used in Chinese medicine (Ou and Kwok, 2004; Cheung et al., 2006; Lerma-García et al., 2009; Karlen et al., 2020). Nonetheless, the production of these acids is limited by the amount of hydroxycinnamate esters in the feedstock, by the plant productivity, and by the extraction and purification processes. Using even part of the massive agroindustry grass biomass leftovers to isolate these phenolic acids could significantly decrease the costs of production (Karlen et al., 2020).

Grass cell walls are excellent sources of FA and *p*CA (Harris and Hartley, 1980; Hatfield et al., 2017). Glucuronoarabinoxylans (GAX) are mainly substituted with FA on arabinosyl units (FA-Ara), although *p*CA linked to arabinosyl units (*p*CA-Ara) has also been found at lower levels (Mueller-Harvey et al., 1986; Lapierre et al., 2018). In contrast, *p*CA is predominantly ester-linked to lignin, mostly to S units (Ralph et al., 1994a; Hatfield et al., 2009; Ralph, 2010). Among widely cultivated crops, *Saccharum* spp., sorghum, and maize are particularly good sources of *p*CA, as they have high levels of *p*CA in their culms, ranging from 3 to 6% of the dry weight, predominating over (releasable) FA (0.5–2% weight) (Masarin et al., 2011; del Río et al., 2015; Hatfield et al., 2017; Fanelli et al., 2020). Furthermore, these are C4 grasses, with highly efficient photosynthetic rates, having great potential as bioenergy crops (Carpita and McCann, 2008; van der Weijde et al., 2013; Hoang et al., 2015).

In biorefinery designs, commonly used pretreatment methods are autohydrolysis, acid catalysis, or alkaline processes (Galbe and Wallberg, 2019). When these treatments are applied to grass biomass, such as sugarcane bagasse or corn stover, part of the ester-linked *p*CA and FA is released into the pretreatment liquors (Gómez et al., 2014; van der Pol et al., 2015). Nevertheless, purification of *p*CA and FA from these pretreatment liquors requires high-cost processes that are especially complex when both acids are present together (Saboe et al., 2018; Karlen et al., 2020). Genetically engineered feedstocks with increased levels of one hydroxycinnamate in preference to the other are therefore highly desirable (Karlen et al., 2020). In this context, there is considerable potential in targeting high levels of ester-linked *p*CA onto hemicelluloses, as it has been shown in grasses that an increase in GAX-*p*CA is usually accompanied by a decrease in FA (Hartley, 1972; Bartley et al., 2013; Li et al., 2018). Another advantage of diminishing FA levels is improving biomass digestibility by reducing cell wall recalcitrance (Himmel et al., 2007), as some grass cell wall components are cross-linked by ferulates (Grabber et al., 1998a, 2000; Ralph et al., 1998, 2004; Ralph, 2010; Hatfield et al., 2017).

The genes encoding enzymes involved with hydroxy-cinnamate incorporation into grass cell walls belong to a specific clade, namely, the Mitchell clade, of the BAHD acyltransferases family (Mitchell et al., 2007; Cesarino et al., 2016; Zhong et al., 2019). Phylogenetic analysis revealed 20 rice genes in this clade, which were named *AT1* to *AT20* (AT = acyltransferase) (Bartley et al., 2013). Expression levels of genes belonging to this clade from several grasses have been associated with *p*CA and FA levels in lignin and GAX. It has been shown that AT3 and AT4 enzymes (also known as PMT) have a *p*-coumaryl-CoA monolignol transferase activity and are involved with *p*CA incorporation into lignin in rice, maize, and *Brachypodium* (Withers et al., 2012; Marita et al., 2014; Petrik et al., 2014). More recently, it was shown that an increase in expression levels of *OsAT5* (*OsFMT*) was followed by an increase in FA specifically ester-linked to lignin in rice (Karlen et al., 2016). Regarding hydroxycinnamate incorporation into hemicelluloses, silencing of *AT9* resulted in decreased FA-Ara levels in *Setaria viridis* and sugarcane cell walls (de Souza et al., 2018, 2019), and there is evidence that *BdAT1* expression levels are also associated with FA content in *Brachypodium* cell walls (Buanafina et al., 2016). Nonetheless, more recently, a study involving the suppression of *SvAT1* in *S. viridis* has evidenced a main role for this gene in the *p*-coumaroylation of arabinose in xylans. The silenced lines had a significant decrease in *p*CA-Ara levels, but also exhibited a small decrease in FA-Ara content in leaves, suggesting that SvAT1 enzyme could participate in GAX feruloylation as well, to a lesser extent (Mota et al., 2020). Furthermore, overexpression of a rice gene (*OsAT10*) influenced *p*CA levels in rice, resulting in *p*CA increase and FA decrease in xylans from mature straw (Bartley et al., 2013). A similar effect was observed for the heterologous overexpression of *OsAT10* in switchgrass, which resulted in increased levels of total ester-linked *p*CA, but only in the green leaves (Li et al., 2018). Recently, an ortholog of *OsAT10* in barley, *HvAT10*, was shown to influence the *p*CA and FA levels in the whole grain (Houston et al., 2020). Therefore, there is considerable potential in manipulating the expression of the Mitchell clade genes to generate biomass feedstocks that are simultaneously less recalcitrant and more suitable for hydroxycinnamic acid generation in biorefineries. In this context, manipulating *AT10* genes seems suitable for *p*CA production.

Although BAHD acyltransferases enzymes were shown to be involved with hydroxycinnamate incorporation in grasses, several aspects still need to be investigated, especially in large-statured crops, such as sugarcane and maize. For sugarcane, the gene encoding an enzyme related to *p*CA incorporation into its GAX is yet to be identified and characterized. This would allow a better understanding of *p*CA incorporation into grass cell walls and could have applications in biorefineries, in which both biofuels and this high-valued chemical could be produced. In this work, genomic, transcriptomic, and expression analyses permitted to identify sugarcane genes belonging to the Mitchell clade. Overexpression of sugarcane *ScAT10*, an ortholog of *OsAT10*, in maize allowed to functionally characterize this gene, which affected hydroxycinnamate levels of arabinoxylan hemicelluloses increasing up to 10 times the total *p*CA/FA ratio in culms.

## Materials and Methods

### Identification, Annotation, and Phylogenetic Analysis of Mitchell Clade BAHD Family Members

To identify putative sugarcane BAHD acyltransferases from the Mitchell clade, we searched in different databases of both genomic and transcriptomic sequences. Two sugarcane genomes were used, from cultivars SP80-3280 (Riaño-Pachón and Mattiello, 2017) and R570 BAC (bacterial artificial chromosome), as well as R570 STP (single tilling path) (Garsmeur et al., 2018). Transcriptomics data used were SUCEST (Sugarcane EST project) dataset (Vettore et al., 2003; Vicentini et al., 2012) and RNA-seq from internodes (Vicentini et al., 2015) and leaves (Cardoso-Silva et al., 2014). We performed a BLASTp (Altschul et al., 1990) search into predicted protein databases, using Mitchell clade sequences from other grasses as a query (Bartley et al., 2013). For transcriptomic databases that consisted of DNA sequences only, we performed BLASTn search, and the DNA annotated sequences were then translated to protein using the Expasy translate tool^1^. To obtain higher sensitivity in the identification of sequences belonging to the BAHD acyltransferases family, we also conducted HMMER searches in the above-mentioned datasets, using hmmsearch and jackhmmer algorithms (Eddy, 2011). Furthermore, we updated the annotation of sorghum Mitchell clade proteins, performing BLASTp and HMMER searches into a more recent version of the genome, *S. bicolor 313 v3.1* (McCormick et al., 2018), which was downloaded from Phytozome 12^2^. The proteins containing the Pfam transferase domain (PF02458) were annotated and aligned with previously published proteins in the Mitchell clade from rice, maize, *Brachypodium*, *S. viridis*, and *Arabidopsis* (Bartley et al., 2013; de Souza et al., 2018). A maximum likelihood phylogenetic analysis was performed using PhyML 3.0 (Guindon et al., 2010). Parameters were JTT + G + F substitution model, selected using smart model selection (Lefort et al., 2017), and aLRT as the test method. The phylogenetic tree was visualized using the iTOL software^3^. After identification of sugarcane and sorghum proteins belonging to the Mitchell clade, a new phylogeny of specifically this clade was performed, selecting one sugarcane representative sequence for each ortholog group, using the same parameters. Proteins identified as belonging to the *AT10* sub-clade were aligned using ClustalW, and an identity matrix was generated in the BioEdit^®^ software. We also analyzed the expression profile of *AT10* orthologs in rice (Jain et al., 2007), *Brachypodium* (Winter et al., 2007; Sibout et al., 2017), and maize (Hoopes et al., 2019) using the eFP Browser tool^4^.

### Mitchell Clade Gene Expression Analysis in Sugarcane H321 by RT-qPCR

Sugarcane hybrids (Masarin et al., 2011) were cultivated from May 2015 to May 2016, in an experimental field located at Lorena, São Paulo, Brazil (22°43′51″ S, 45°07′29″ W). Plants were grown in 0.60 m × 1.0 m rows with 3.0 m × 2.0 m spacing between hybrids. Culms from 1-year-old first ratoon hybrid H321 plants were harvested. For each culm, the first internode (from top to bottom), in which superior and inferior nodes were clearly identified, was sampled as previously described (Collucci et al., 2019). These internodes were cut in 25 mm circles. The first 2 mm external section, containing epidermal cells, was removed. The remaining material was divided into three fractions corresponding to the rind, pith–rind interface, and pith (Costa et al., 2013). All leaves were sampled from 3-month-old H321 sugarcane plants, collected in 2015. The harvested tissues were immediately frozen in liquid nitrogen and then stored at −80°C until use. All samples were ground in a cryogenic grinder (2010 Geno/Grinder^®^, Spex SamplePrep^®^), always keeping the material in touch with liquid nitrogen. RNA was extracted using Concert™ Plant RNA Reagent (Invitrogen™). RNA quantity and quality were accessed using Biodrop duo (Biochrom), whereas integrity was checked with electrophoresis in 0.8% agarose gel. RNA samples were treated with DNAse RQ1 RNAse-free DNAse^®^ (Promega Co.), and the first-strand cDNA was synthesized using SuperScript III Reverse Transcriptase^®^ (Invitrogen^®^) following the manufacturer’s instructions. Primers specific for each of the nine sugarcane genes identified in the Mitchell clade were designed in the last exon and 3′UTR region, using both genomic and transcriptomic sequences (Supplementary Table 1), except for the *GAPDH* reference gene previously characterized (Bottcher et al., 2013). All quantitative real-time PCR (qRT-PCR) reactions were conducted in 96-well plates with SYBR™ GREEN PCR master mix (Applied Biosystems™, ThermoFisher Scientific™), in a 7,500 Fast Real-time PCR system (Applied Biosystems™, ThermoFisher Scientific™) using the program 95°C (20 s)/95°C (3 s)/40 cycles 60°C (30 s). Primer specificity was accessed through melting curve analysis, with program 95°C (20 s)/60°C (1 min)/95°C (15 s)/60°C (15 s) (Supplementary Figure 1A). For expression quantification, the efficiency of each primer pair was estimated using the LinRegPCR software (Ramakers et al., 2003; Ruijter et al., 2009; Supplementary Table 2A). Relative expression was calculated including efficiency correction and normalization with reference genes (Pfaffl, 2001; Vandesompele et al., 2002), using the Relative Quantification software (Thermo Fisher Cloud™).

### Generation of *Ubi:ScAT10* Maize Lines

A plant transformation construct whereby *ScAT10* would be expressed from the strong constitutive maize ubiquitin promoter was made as follows. The identified *ScAT10* coding sequence (1362 bp) flanked by *attb1*/*attb2* recombinant regions (25 bp each) was synthesized in the puc57 vector (FASTBIO). Following Gateway cloning^®^ instructions (Invitrogen™), a PCR product consisting of *ScAT10* CDS flanked by *attb* regions was generated (Supplementary Table 1), gel purified, and recombined with plasmid *pDONR221* in a BP reaction (BP clonase, Invitrogen™). The generated entry vector was confirmed by restriction enzyme digestion (New England BioLabs™) and recombined with destination vector *Ox:pzp221b* (Ox—overexpression) (Marita et al., 2014), through LR reaction (LR clonase, Invitrogen™). The resulting *pzp221b:Ox:ScAT10* was confirmed by restriction enzyme digestion, and *ScAT10* CDS insert was confirmed by sequencing (Eurofins) (Supplementary Figure 2). Maize HiII lines were transformed with the *pzp221b:Ox:ScAT10* construct, using *Agrobacterium tumefaciens*, at the Plant Transformation Facility, Iowa State University^5^, following their standard procedures. Ten biological independent transgenic events were received and regenerated in Petri dishes with selective media containing Bialaphos. These plantlets were transplanted to 5 cm pots in a growth chamber with a 24 h light regime. After a month, they were transplanted to larger pots (11.5 liters) in a greenhouse with a 16 h light regime (Supplementary Figure 3). Plants were periodically watered and fertilized with soluble fertilizer (nitrogen–phosphorus–potassium 18–2–18). The 10 independent lines were further analyzed, both genotypically and phenotypically. DNA was isolated from the 4^th^ leaf (before complete expansion) of transgenic plants after a month of growth, as previously described (Marita et al., 2014). PCR genotyping was conducted using specific primers targeting *ScAT10* (Supplementary Table 1). To avoid annealing with endogenous maize *ZmAT10* genomic sequences, we designed primers in the exon–exon junction, specifically targeting the inserted sugarcane CDS (Supplementary Figure 4).

### Maize *Ubi:ScAT10* Lines Gene Expression Levels by RT-qPCR

The base of the first fully developed leaf after ear development was harvested from 3-month-old greenhouse-grown plants. RNA was extracted from these tissues using Spectrum™ plant total RNA kit (protocol A; Sigma Aldrich^®^). RNA quantity/quality was inferred *via* spectrophotometry, and integrity was checked by electrophoresis on a 0.8% agarose gel. One microgram of RNA was treated with RNAse-free DNAse (Promega Co.). First-strand cDNA was synthesized with GoScript Reverse Transcription System (Promega Co.), using 400 ng of DNAse-treated RNA and OligodT primers, following the manufacturer’s instructions. For RT-qPCR analysis, primers were designed for target gene *ScAT10*, as well as for reference genes *ZmLUG* and *ZmMEP* (Manoli et al., 2012) using Primer3plus (Supplementary Table 1). *ScAT10* primers were designed in a region encompassing part of *ScAT10* CDS and the T-DNA insert before the NOS transcriptional termination site (Supplementary Figure 2), conferring specificity. Maize reference gene primers were designed in the last exon and 3′UTR region. All RT-qPCR reactions and relative expression calculations were conducted as described above (Supplementary Figure 1B and Supplementary Table 2B).

### Plant Cell Wall Isolation for Chemical Compositional Analysis

The whole culms of 4-month-old senescent plants (age determined as after transferring from tissue culture) were harvested. Samples were ground in a UDY mill (Udy Corp., Fort Collins, CO., United States) with a 1 mm screen and dried overnight at 55°C. Cell wall was extracted as previously described (Hatfield et al., 2009). Ground tissue (5-10 g) was incubated in Nalgene centrifuge bottles with 50 mM tris acetate buffer (pH 6), shaken, and centrifuged (6,500 rpm for 20 min) three times. Samples were then extracted three times with 80% ethanol, using the same procedure. Pellets were extracted another three times with acetone and one time with chloroform:methanol (2:1 v/v). Samples were then washed with acetone to remove the chloroform:methanol mix. Final cell wall residues were air-dried in a fume hood, fully dried, and stored in a 55°C oven until further use for assays.

### Ester-Linked *p*CA and FA Released by Mild Alkaline Treatment

The dried cell walls (100 mg) were incubated in 3 ml vials with 100 μl of 1 mg/l 2-hydroxycinnamic acid (*o*CA) as internal standard and 2.4 ml 2 M NaOH, at room temperature for about 20 h (Grabber et al., 1995). Samples were acidified to pH 2 with 12.1 M HCl. Phenolics were then extracted three times with 2.0 ml diethyl ether. Released phenolics were identified and quantified as trimethylsilyl derivatives (40 μl TMSI and 10 μl pyridine) by GLC-FID (Agilent Technologies 7890 GC system) on a ZB-1 column (Phenomenex, Zebron 100% dimethylpolysiloxane; 30 m × 0.25 mm, 0.25 μm film). The GLC conditions were injector 315°C, detector 300°C, and a temperature program of 200°C 1 min and 4°C min^–1^ to 248°C held for 2 min, followed by 30°C min^–1^ to 300°C before holding for 20 min. All GC temperature programs were run at 20 psi constant pressure and split ratio 30:1. Standards of FA and *p*CA were used to identify and quantify phenolic products.

### Mild Acid Hydrolysis

The dried cell walls were treated by mild acidolysis according to a previous report (Lapierre et al., 2018). Hydrolysates were analyzed by HPLC. For this, the samples were quantified on a Shimadzu Nexara X2 HPLC equipped with a Phenomenex Kinetex C18 column (2.6 μm × 2.1 mm × 150 mm, P/N: 00F-4462-AN). The mobile phase was a binary gradient of solvent A: water + 0.1% formic acid and solvent B: acetonitrile + 0.1% formic acid. The detector was a photo-diode array scanning from λ = 250–600 nm, quantification was performed at λ = 270 nm, and a 5-point calibration curve was determined using authentic MeAra-*p*CA, MeAra-FA, and *o*CA (54 μg/ml, internal standard).

### Cell Wall Total Neutral Sugars

Total neutral sugars in the cell walls were released according to a previous report (Rancour et al., 2012). Monosaccharides were converted to their alditol acetate derivatives following the protocol described by Blakeney et al. (1983). Sugar derivatives were quantified by GLC-FID on a Shimadzu GC-2010 using a 007-225 methylpolysiloxane column (30 m × 0.25 mm with 0.25 μm film thickness; Quadrex Corporation). GLC conditions were injector 220°C, detector 240°C, and a temperature program of 215°C for 2 min, 4°C min^–1^ to 230°C before holding for 11.25 min run at a constant linear velocity of 33.4 cm.s^–1^, and split ratio 25:1.

### Derivation Followed by Reductive Cleavage (DFRC)

The derivatization followed by reductive cleavage (DFRC) analysis was performed on maize samples as described for the optimized DFRC protocol (Regner et al., 2018). The maize sample (50 mg) was stirred in a two-dram vial, with PTFE pressure release cap (Chemglass CG-4912-02), in acetyl bromide/acetic acid (1/4 v/v, 3 ml) at 50°C for 2.5 h. The solvent was removed on a SpeedVac (Thermo Scientific SPD131DDA, 50°C, 35 min, 1.0 Torr, 35 Torr/min). The crude film was suspended in absolute ethanol (0.5 ml), and the ethanol was then removed on the SpeedVac (50°C, 15 min, 6.0 Torr, 35 Torr/min). The residue was suspended in 1,4-dioxane:acetic acid:water (5/4/1 v/v/v, 5 ml), and nano-powder zinc (150 mg) was added. The reaction was then sealed and sonicated for 1 h at room temperature. The reaction was spiked with a mixture of isotopically labeled internal standards (H-d_8_, G-d_8_, S-d_8_, G-DD*p*CA-d_10_, S-DD*p*CA-d_10_, G-DDFA-d10, and S-DDFA-d_10_) and quantitatively transferred using dichloromethane (DCM, 2 × 2 ml) to a separatory funnel charged with saturated ammonium chloride (10 ml). The organics were extracted with DCM (3 ml × 10 ml), combined, dried over anhydrous sodium sulfate, and filtered through qualitative filter paper, and the solvent was removed on a rotary evaporator (water bath at <50°C). The free hydroxyl groups were then acetylated for 30 min in pyridine and acetic anhydride (1/1 v/v, 5 ml), after which the solvent was removed by rotary evaporation to give a crude oily film. To remove most of the polysaccharide-derived products, the crude DFRC product was loaded onto an SPE cartridge (Supelco Supelclean LC-Si SPE tube, 3 ml, P/N: 505048) with DCM (3 ml × 1.0 ml). The products were eluted with n-hexane:ethyl acetate (1:1, v:v, 8 ml), and the combined solvents were removed on a rotary evaporator and transferred with DCM to a GC–MS vial for a final sample volume of ∼1 ml. The samples were analyzed on a triple-quadrupole GC–MS/MS (Shimadzu GCMS-TQ8030) operating in multiple-reaction-monitoring (MRM) mode using synthetic standards for authentication and calibration. Calibration curves were determined from the ratio of peak areas of the synthetic *trans*-product to the corresponding isotope-labeled internal standard vs. the ratio of their concentrations.

### Enzymatic Digestibility of *Ubi:ScAT10* Maize Lines

Approximately 50 mg of cell wall material was suspended in buffer [30 mM citrate/NaOH pH 4.5, 0.01% (w/v) NaN_3_] containing 5 U/ml of cellulase (Celluclast, Novozymes^®^) and 5 U/ml xylanase (Sigma-Aldrich, St. Louis, MO, United States), adjusting the volume to 1 U/mg of cell wall. Samples were incubated at 40°C in a shaker for 48 h. Tubes were centrifugated, and an aliquot of the supernatant was analyzed for total carbohydrate using the phenol-sulfuric method (DuBois et al., 1956). Percent of conversion was estimated by the ratio of sugars released and the sum of xylose, arabinose, and glucose in the cell walls determined for each event.

### Statistical Analysis

For sugarcane expression profiling, RT-qPCR experiments were performed in technical triplicate for each biological duplicate, and statistically significant differences were determined using the unpaired Student’s *t*-test (α = 0.05). For maize transgenics characterization, RT-qPCR and mild acidolysis were performed in technical triplicate, whereas determination of ester-linked phenolics released by mild alkaline treatment, total cell wall neutral sugars, DFRC, and digestibility analysis was performed in technical duplicate. Statistically significant differences were determined using the unpaired Welch’s *t*-test (α = 0.05) comparing the mean of analyzed biological individuals for each group (*Ubi:ScAT10* transgenic lines, *n* ≥ 4 and B73/empty vector control lines, *n* ≥ 3). All statistical analyses were conducted in R^6^.

## Results

### Identification and Phylogenetic Analysis of BAHD Family Members From Mitchell Clade A

**FIGURE 1:**
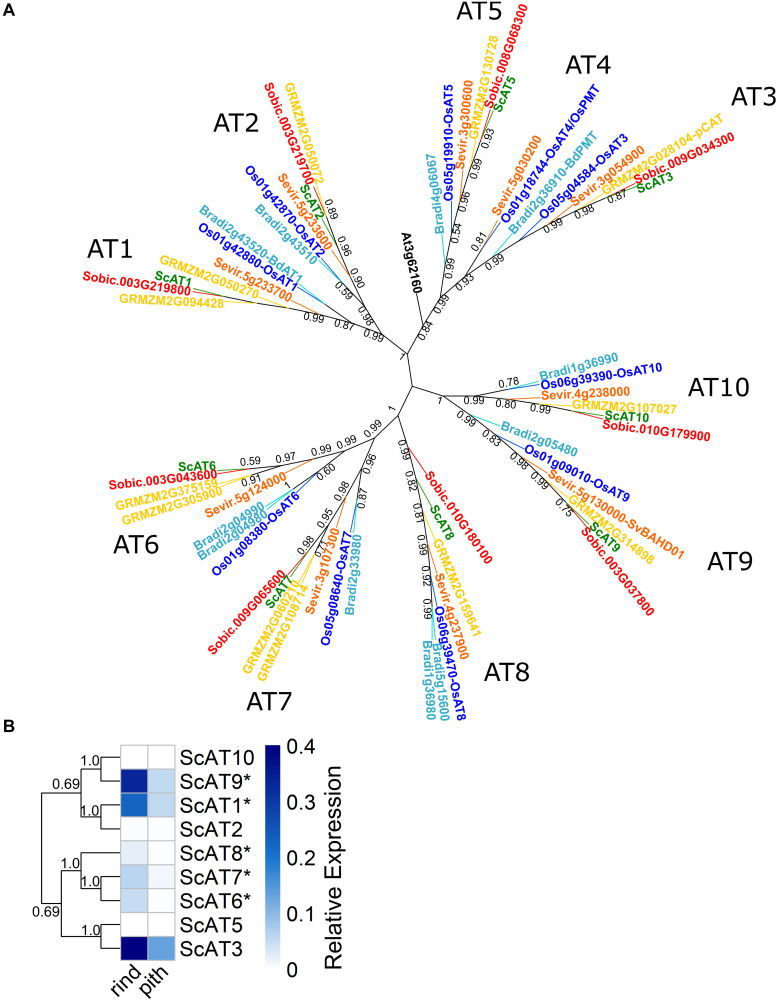
Evolutionary relationship and expression profile of sugarcane Mitchell clade A genes. **(A)** Unrooted maximum likelihood phylogeny of sugarcane, related grasses, and *Arabidopsis*. Topology support shown was obtained with the aLRT method, and values below 0.4 were not represented. Species and branches are colored in dark green (*Saccharum* spp.), red (*Sorghum bicolor*), yellow (*Zea mays*), orange (*Setaria viridis*), light blue (*Brachypodium distachyon*), dark blue (*Oryza sativa*), and black (*Arabidopsis thaliana*). **(B)** Heatmap of RT-qPCR relative expression levels of Mitchell clade A genes in sugarcane rind and pith sections of a young internode. Dendrogram represents a neighbor-joining phylogenetic analysis of the sugarcane genes, with topology support shown as the ratio of bootstrap runs. Relative expression was normalized with the reference gene *ScGAPDH* (2^–ΔCt^) (Pfaffl, 2001). Values with * were significantly different between tissues by Student’s t-test, *p* < 0.05.

We used a bioinformatics approach to identify sugarcane genes belonging to the Mitchell clade A (Mitchell et al., 2007; Molinari et al., 2013) of BAHD acyltransferases. BLAST (Altschul et al., 1990) and HMMer (Eddy, 2011) searches against genomic and transcriptomic sugarcane datasets, followed by a phylogenetic analysis for sugarcane, sorghum, *Brachypodium*, *Setaria*, rice, maize, and *Arabidopsis* (Figure 1A) revealed nine sugarcane unigenes in this clade with complete open reading frames (ORFs), which were named as *ScAT1* to *ScAT10* lacking of *ScAT4*, based on the nomenclature of Bartley et al. (2013) (Supplementary Tables 3A,B). We constructed a phylogenetic tree in which, for each sub-clade containing *AT1* to *AT10* ortholog genes, only one sugarcane identified sequence (unigene) was represented, prioritizing those from R570 and SP8032-80 genomes whenever possible (Figure 1A and Supplementary Tables 3A,B). Nonetheless, for the sub-clade containing *AT7* orthologs, only sugarcane transcript sequences were identified in the analyzed datasets (Supplementary Table 3B). Therefore, the currently available sugarcane genomic sequences do not yet cover the full transcriptome for this crop, and thus RNA-seq remains a powerful tool to provide additional information for the identification of new genes in sugarcane.

### Expression Analysis of Sugarcane Genes From the Mitchell Clade A

To investigate the expression profile of sugarcane genes identified in Mitchell clade A, we analyzed EST data from distinct libraries of SUCEST (Vettore et al., 2003; Vicentini et al., 2012; Supplementary Figure 5). All nine genes were expressed, indicating that they are functional in at least one tissue at a stage of sugarcane development. In the internode tissues, *ScAT3*, *ScAT8*, *ScAT9*, and *ScAT1* had the highest levels of expression. All these genes were also expressed in a reproductive (inflorescence) stage. On the other hand, *ScAT5* and *ScAT10* showed low and restricted expression in root and inflorescence tissues, respectively.

Considering the importance of sugarcane bagasse as a feedstock for biorefineries, RT-qPCR analysis was conducted in culm tissues. We selected the young internode one (below the uppermost node) from a hemicellulose-rich sugarcane hybrid H321 (Masarin et al., 2011), which was separated into the rind (outermost part) and pith (inner part) regions. It has been shown that the sugarcane rind and pith have distinct chemical compositions, with the rind having more lignin and hemicellulose than the pith (Costa et al., 2013). Overall, five genes (*ScAT1*, *ScAT6*, *ScAT7*, *ScAT8*, and *ScAT9*) had significantly higher expression levels (*p* < 0.05) in the rind than in the pith (Figure 1B). The predominant cell types in the rind are vessels and sclerenchyma fibers, whereas in the pith, parenchyma cells are more abundant (Costa et al., 2013; Ferraz et al., 2014). The vessels and fibers present a higher content of hydroxycinnamates and lignin (Siqueira et al., 2011). Therefore, a higher expression in the rind corroborates the hypothesis that the enzymes encoded by these genes are related to FA or *p*CA incorporation into secondary cell walls.

Both *ScAT10* and *ScAT5* expressions, on the other hand, were not detected in the internode (Figure 1B), as found for EST data. However, they were expressed in young sugarcane leaves at low levels (Supplementary Figure 6). This expression pattern of *ScAT10* supports a potential role of this gene in *p*CA incorporation into hemicelluloses, considering that *p*CA-Ara was detected at low levels in cell walls from grasses and in a higher proportion in leaves than in internodes (Mueller-Harvey et al., 1986; Lapierre et al., 2018).

### Protein Identity and *In silico* Expression Pattern of *AT10* Orthologous Genes

The sugarcane ScAT10 full-length amino acid sequence shares a high percentage of identity with the AT10 amino acid sequences of *Brachypodium* (79%), rice (80%), *Setaria* (87%), maize (86%), and sorghum (93%) (Supplementary Table 4A). In the transferase domain region, which contains motifs associated with BAHD acyltransferase function, the degree of identity is even higher, having more than 81% identity with proteins in the C3 photosynthetic pathway species (rice and *Brachypodium*) and more than 90% for C4 species (sorghum, maize, and *Setaria*) (Supplementary Table 4B and Supplementary Figure 7). This suggests that AT10 enzymes may have a similar role in a variety of C3 and C4 plant species.

We analyzed the *AT10* orthologs’ expression patterns among different organs and tissues *in silico* using available data with the eFP browser tool (see text Footnote 4) (Winter et al., 2007) for rice, *Brachypodium*, and maize (Supplementary Figure 8). For rice, *OsAT10* expression levels were higher in the inflorescence (Supplementary Figure 8A; Jain et al., 2007), similar to sugarcane EST data (Supplementary Figure 5). In *Brachypodium*, expression was higher in young leaf tissue, root, and young internode (Winter et al., 2007; Sibout et al., 2017; Supplementary Figure 8B). For maize, *ZmAT10* showed higher expression in one specific immature leaf stage, followed by meiotic tassel and seeds in different stages (Hoopes et al., 2019; Supplementary Figure 8C). Similarly to *ScAT10* (Figure 1B and Supplementary Figure 5), the expression pattern of *OsAT10* and *ZmAT10* consisted of relatively high expression levels in less lignified tissues, such as reproductive organs and immature leaves (Jung and Casler, 2006; Rancour et al., 2012; Bottcher et al., 2013), and lower or no expression in analyzed internodes (Supplementary Figures 8A,C). This profile provided further evidence that AT10 enzymes may share a conserved role, at least in rice, maize, and sugarcane. This expression pattern is also consistent with this AT10’s role in *p*CA levels of hemicellulose, and not lignin, as already shown for OsAT10.

*ScAT10* is, therefore, a strong candidate for studies aiming at functional characterization of genes encoding enzymes involved with cell wall *p*-coumaroylation. Taken together, the undetected *ZmAT10* expression in internodes, maize’s close evolutionary relationship with sugarcane, and its relative ease of transformation (Joyce et al., 2010), all make maize a suitable plant for *ScAT10* functional characterization.

### Overexpression of *ScAT10* in Maize Lines

**FIGURE 2:**
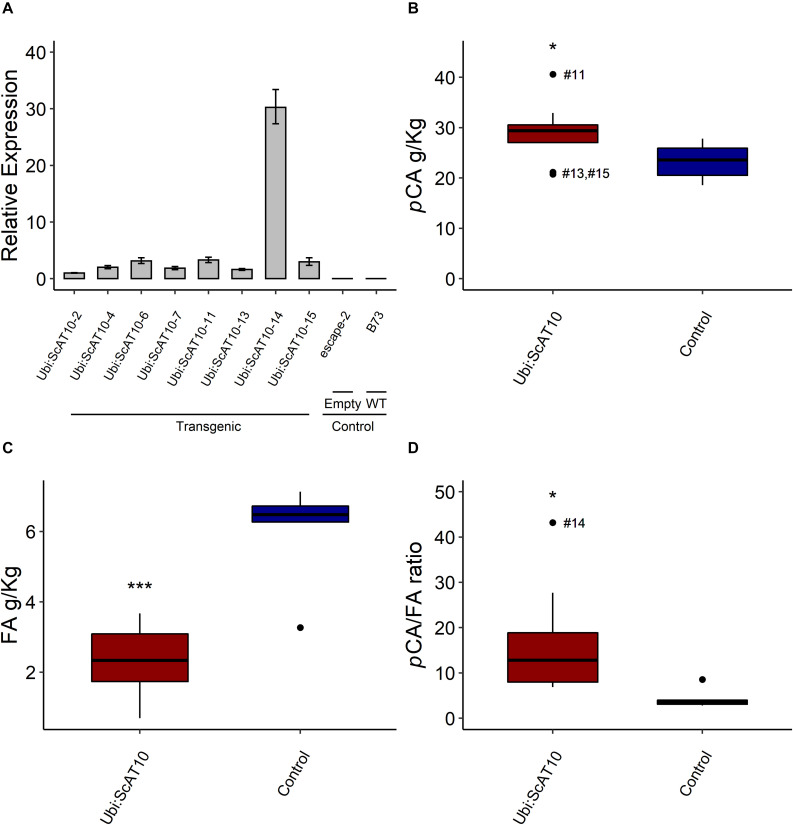
Impact of overexpressing *ScAT10* in maize lines. **(A)** Relative expression levels of *ScAT10* gene in maize independent transgenic lines (*Ubi:ScAT10*), empty vector lines *escape-2* and *escape-3*, and wild-type (WT) B73. Expression levels calculated by the ΔΔCt method (Pfaffl, 2001; Vandesompele et al., 2002) using *ZmMEP* and *ZmLUG* as reference genes and *escape-2* as the reference sample. Columns represent the means, and error bars represent the superior and inferior limits determined by standard deviation (*n = 3* technical replicates). **(B)** *p*-Coumaric acid content (*p*CA), **(C)** Ferulic acid (FA) content, and **(D)** Ratio of pCA/FA content from culms, released by mild alkaline treatment. Red boxes represent the distribution of values for transgenic lines (*Ubi:ScAT10-2*, *-4*, *-6*, *-7*, *-11*, *-13*, *-14*, and *-15*, total *n = 8* plants). Blue boxes represent the distribution of values for control lines (empty vector *escape-2* and *escape-3* and wild-type (WT) B73, total *n = 6* plants, one for each empty vector and four B73). Each box delimits values within the first and third quartile ranges. The horizontal black lines in the boxes represent the median values. Whiskers represent the data range. Outliers were represented by black dots, and the #numbers identify the number of the line for *Ubi:ScAT10* lines. * represents the significant difference at *p* < 0.05, and *** represents the significant difference at *p* < 0.001 between the mean of transgenics and the mean of controls using Welch’s *t*-test. See Supplementary Table 5 for actual values for each line.

To conduct the functional characterization of *ScAT10*, maize HiII lines were transformed with the sugarcane *ScAT10* full-length cDNA under control of the maize ubiquitin promoter (Supplementary Figure 2), generating 10 independent lines, 8 confirmed as positives for the presence of the transgene (named with prefix *Ubi:ScAT10* followed by *-2*, *-4*, *-6*, *-7*, *-11*, *-13*, *-14*, and *-15*) and 2 negatives for the presence of the transgene (named *escape-2* and *escape-3* empty vectors), which were used together with wild-type B73 as controls in further analyses (Supplementary Figure 9). RT-qPCR in the first leaf blade above the ear of maize plants showed a high expression level of *ScAT10* in all positive transgenic lines, especially *Ubi:ScAT10-14*, whereas the controls wild-type and escape lines did not show expression of *ScAT10* (Figure 2A). We also analyzed the expression levels of the endogenous *ZmAT10* in both transgenic and control lines, and no expression was detected, confirming data from eFP analysis (Supplementary Figure 8C).

### Impact of *ScAT10* Overexpression on Maize Cell Wall Hydroxycinnamate Content

To assess the impact of the overexpression of *ScAT10* in maize cell walls from mature stems, we first measured ester-linked *p*CA and FA released by mild alkaline treatment (Figure 2 and Supplementary Table 5). The comparison of the mean values of *Ubi:ScAT10* transgenic lines (29.13 g/kg) with the mean of control lines (23.30 g/kg) using a Welch’s t-test showed a significant increase in *p*CA content (*p* = 0.027), corresponding to an increase of 75% for *Ubi:ScAT10-11* (Figure 2B). Transgenic lines also showed a significant decrease in FA (*p* = 0.0002), reaching 88% decrease for line *Ubi-ScAT10-14* (Figure 2C). The effect of the overexpression of *ScAT10* on both *p*CA and FA contents can be better visualized by examining the *p*CA/FA ratio, which had a significant increase (*p* = 0.013) when comparing the means of transgenic with control lines (Figure 2D). Independent lines *Ubi-ScAT10-2*, *Ubi:ScAT10-6*, *Ubi:ScAT10-11*, and *Ubi:ScAT10-14*, which had the greatest *p*CA/FA ratios, were selected for further analysis.

### Hydroxycinnamate Content Specifically Attached to GAX or Lignin

**FIGURE 3:**
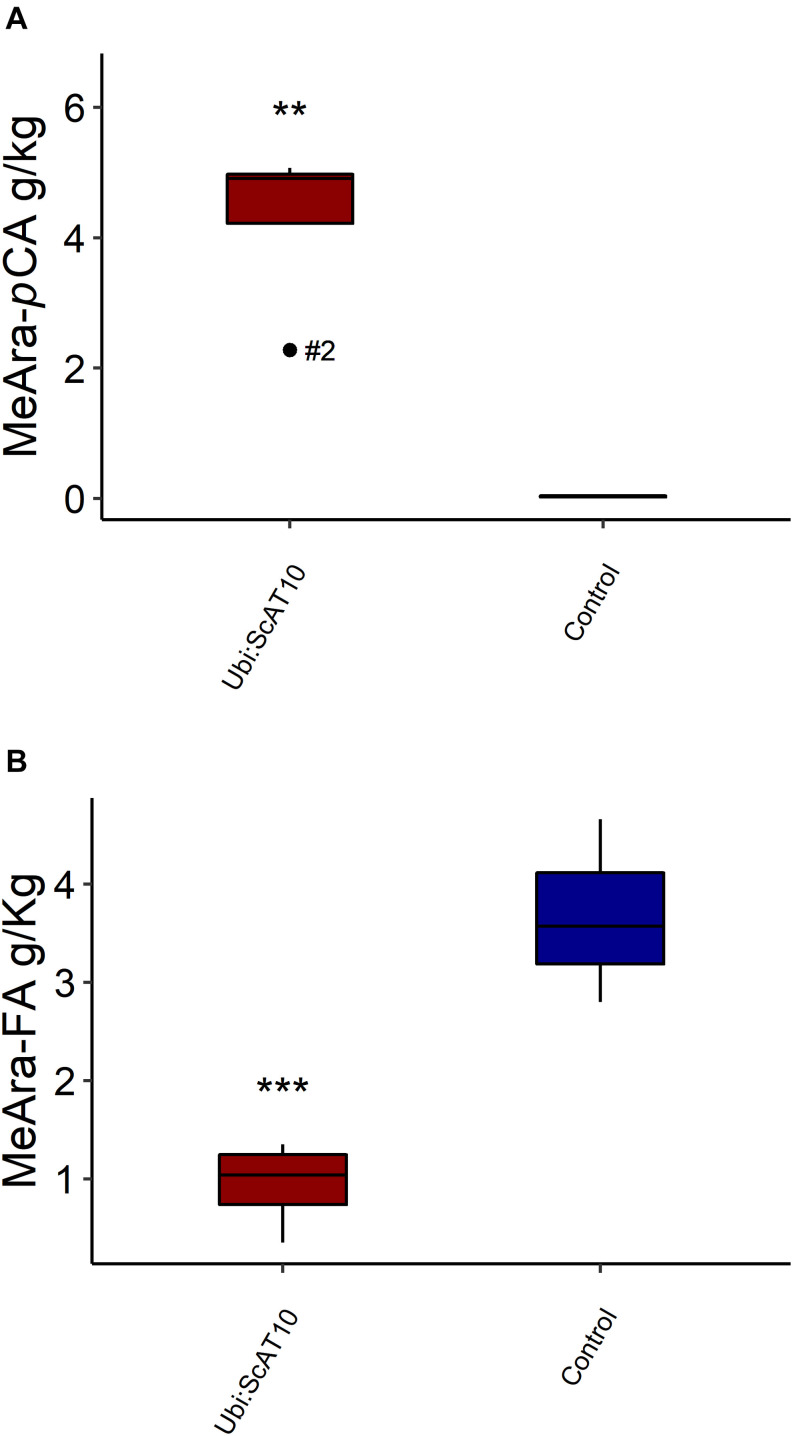
Hydroxycinnamates specifically attached to GAX in culms cell walls, released by mild acidolysis, from transgenic *Ubi:ScAT10* and control lines. **(A)** *p-*Coumaric acid linked to arabinose (*p*CA-Ara). **(B)** Ferulic acid linked to arabinose (FA-Ara). Red boxes represent the distribution of values for transgenic lines (*Ubi:ScAT10-2*, *-6*, *-11*, and *-14*, total *n* = 4 plants). Blue boxes represent the distribution of values for control lines (empty vector *escape-2* and *escape-3* and wild-type (WT) B73, total *n* = 3 plants). Each box delimits values within the first and third quartile ranges. The horizontal black lines in the boxes represent the median values. Whiskers represent the data range. Outliers were represented by black dots, and the numbers identify the number of the line for *Ubi:ScAT10* lines. ** represents the significant difference at *p* < 0.01, and *** represents the significant difference at *p* < 0.001 between the mean of transgenics and the mean of controls using Welch’s t-test. See Supplementary Table 6 for actual values for each line.

**FIGURE 4:**
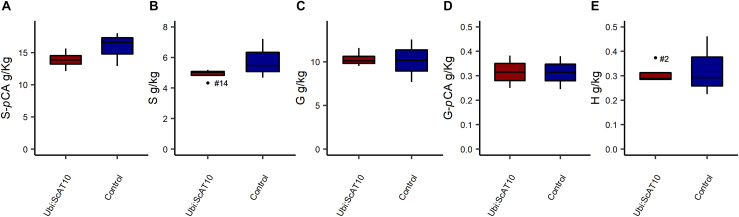
**(A)** S-*p*CA units, **(B)** S non-acylated units, **(C)** G non-acylated units, **(D)** G-*p*CA units, and **(E)** H units amounts in lignin from transgenic *Ubi:ScAT10* and control lines as determined by DFRC in culms cell walls. Red boxes represent the distribution of values for transgenic lines (*Ubi:ScAT10-2*, *-6*, *-11*, and *-14*, total *n* = 4 plants). Blue boxes represent the distribution of values for control lines (empty vector *escape-2* and *escape-3* and wild-type (WT) B73, total *n* = 3 plants). Each box delimits values within the first and third quartile ranges. The horizontal black lines in the boxes represent the median values. Whiskers represent the data range. Outliers were represented by black dots, and the numbers identify the number of the line for *Ubi:ScAT10* lines. See Supplementary Table 7 for actual values for each line.

As *p*CA and FA are attached to both GAX and lignin in grass cell walls (Ralph, 2010; Wilkerson et al., 2014; Karlen et al., 2016; Hatfield et al., 2017), we used two orthogonal methods available to diagnose the hydroxycinnamates on each. To access hydroxycinnamate content specifically from the hemicellulosic component, we used a mild acidolysis procedure (HCl/dioxane) (Lapierre et al., 2018; Figure 3 and Supplementary Table 6). This method cleaves GAX arabinosyl glycosidic bonds while leaving most esters intact, allowing the determination of *p*CA-Ara and FA-Ara contents. Transgenic lines showed a very large (around 160-fold) and significant (*p* = 0.004) increase in *p*CA-Ara (Figure 3A). Overexpression of *ScAT10* resulted in modified cell wall composition, promoting *p*CA incorporation into the xylan, a characteristic that was nearly non-existent in the cell wall from the culms of the wild-type maize. The FA-Ara levels in those same tissues were significantly (*p* = 0.012) reduced in transgenic lines (Figure 3B), which had 75% less FA than controls on average. To determine the fraction of *p*CA specifically attached to lignin, we used the derivatization followed by the DFRC method, which cleaves lignin β-aryl ether bonds and preserves ester bonds, releasing monolignol ester conjugates, such as S-*p*CA (sinapyl *p*-coumarate, from syringyl units) and G-*p*CA (coniferyl *p*-coumarate, from guaiacyl units) (Lu and Ralph, 1999; Regner et al., 2018; Figure 4 and Supplementary Table 7). No significant difference was detected in the means of S-*p*CA levels of transgenic lines and controls (*p* = 0.164), showing that the overexpression of *ScAT10* had no effect on *p*CA content in the lignin. G-*p*CA levels were also not significantly different (*p* = 0.483) and very low in transgenic and control lines, corroborating that *p*CA is mostly attached to S units in grasses as has been noted previously (Ralph et al., 1994a; Hatfield et al., 2009; Ralph, 2010). DFRC data also reveal no significant difference for non-acylated H (*p* = 0.417), G (*p* = 0.453), and S (*p* = 0.176) unit contents, suggesting that *ScAT10* overexpression had no impact on the proportion of non-acylated lignin units. In summary, the perturbation of *p*CA levels on the arabinoxylans had insignificant effect on the lignin component.

### Cell Wall Total Neutral Sugars Content

**FIGURE 5:**
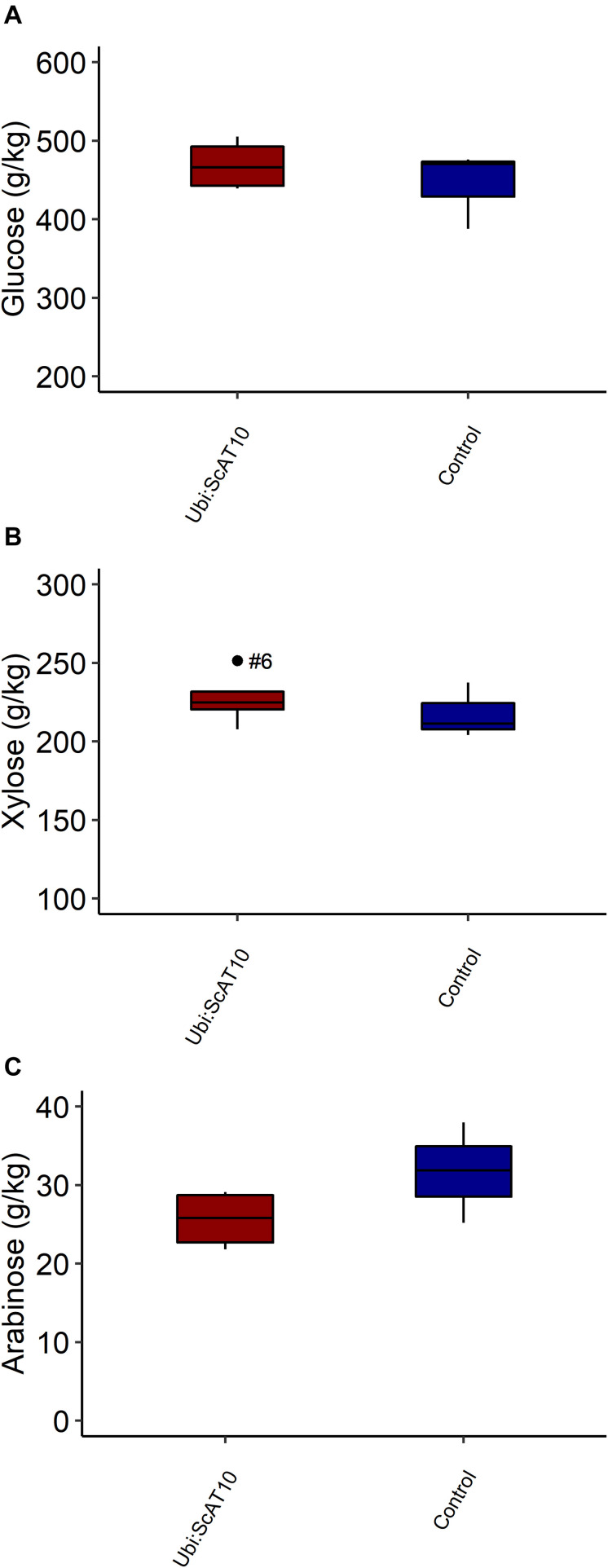
Total neutral sugars in culms cell walls from transgenic *Ubi:ScAT10* and control lines. **(A)** Cell wall glucose content. **(B)** Cell wall xylose content. **(C)** Cell wall arabinose content. Red boxes represent the distribution of values for transgenic lines (*Ubi:ScAT10-2*, *-6*, *-11*, and *-14*, n = 4 plants). Blue boxes represent the distribution of values for control lines (empty vector *escape-2* and *escape-3* and wild-type (WT) B73, total n = 3 plants). Each box delimits values within the first and third quartile ranges. The horizontal black lines in the boxes represent the median values. Whiskers represent the data range. Outliers were represented by black dots, and the numbers identify the number of the line for *Ubi:ScAT10* lines. See Supplementary Table 8 for actual values for each line.

Modification in hydroxycinnamate levels attached to GAX was not accompanied by significant changes in cell wall neutral sugars composition. Xylose and arabinose, the main components of GAX, had similar levels in transgenic and control lines (*p* = 0.258 and *p* = 0.120, respectively). Furthermore, no significant effect was observed in glucose content (*p* = 0.253) (Figure 5 and Supplementary Table 8).

### Enzymatic Digestibility of Maize Lines Overexpressing *ScAT10*

Grass cell wall polymers present cross-linked ferulates and diferulates, forming structures, such as GAX-diFA-GAX and GAX-FA-Lignin (Jung et al., 1983; Ralph et al., 1994b, 1998; Grabber et al., 1998b, 2000). Several studies have shown that the content of FA can be inversely correlated with biomass digestibility (Lam et al., 2003; Bartley et al., 2013; de Souza et al., 2019). Considering the decrease of FA content in maize *Ubi:ScAT10* lines (Figures 2C, 3B), the enzymatic digestibility of the isolated cell walls was investigated (Supplementary Figure 10 and Supplementary Table 9). In all evaluated samples, polysaccharide conversion ranged from 15 to 31%, which is a low digestibility associated with the direct enzymatic digestion of lignified cell walls without pretreatment (Bhutto et al., 2017). Direct *in vitro* digestibility of the transgenic lines did not differ significantly from control, suggesting that the levels of FA decrease observed in the transgenic lines were not enough to affect their digestibility without using sample pretreatment.

## Discussion

In this study, the use of distinct sugarcane genomic and transcriptomic datasets associated with a robust bioinformatics and phylogenetic analysis allowed the identification of nine BAHD acyltransferase sugarcane unigenes that were identified in the Mitchell clade A (Figure 1A), including three (*ScAT10*, *ScAT7*, and *ScAT5*) genes not identified previously (de Souza et al., 2019). Overall, the expression profile of the identified Mitchell clade A sugarcane genes (Figure 1B and Supplementary Figure 5) supports the potential involvement of the enzymes encoded by them in hydroxycinnamate incorporation into cell walls. In both the EST and RT-qPCR data, the genes *ScAT3*, *ScAT9*, and *ScAT1* were highly expressed in internode tissues, whereas *ScAT5* and *ScAT10* were not expressed. A previous study analyzing the expression profile of Mitchell clade A genes in stems of *S. viridis* and *Brachypodium distachyon* (de Souza et al., 2018) showed a similar pattern in these grasses, with *AT9* and *AT3* genes being highly expressed in both species, and very low expression levels found for *AT5* and *AT10*. However, in contrast with the profile in sugarcane, they also found that *SvAT7* had high expression levels in *Setaria* stems, second only to *SvAT9*. This data suggests a few differences among grasses in the expression pattern for some genes in this clade, which could indicate specific function.

In our profiling analysis, *ScAT3* showed high expression in internode tissues, in which secondary cell wall deposition and lignification are high (He and Terashima, 1990; Collucci et al., 2019). These results support a potential role of this gene in *p*CA incorporation into lignin, as observed for its orthologous genes in *Brachypodium* (*BdPMT*), maize (*ZmpCAT*), and their closest homolog in rice (*OsPMT*) (Withers et al., 2012; Marita et al., 2014; Petrik et al., 2014). The gene *ScAT1* was highly expressed in the internode as well as in other vegetative and reproductive tissues, similarly to *ScAT9*. The role of *ScAT9* (*SacBAHD01/AT9*) in feruloylation of xylans has already been demonstrated (de Souza et al., 2019), and *ScAT1* could have a similar role, which was suggested for its *Brachypodium* ortholog *BdAT1* (Buanafina et al., 2016). Nonetheless, it has been recently shown that the ortholog in *S. viridis*, *SvAT1*, has a role in *p*-coumaroylation of GAX, similarly to rice *OsAT10* and sugarcane *ScAT10* (Bartley et al., 2013; Mota et al., 2020). The study also predicted that BdAT1 enzyme has higher affinity for FA, differently from SvAT1, which could accept both *p*CA and FA, suggesting that these enzymes are functionally distinct, despite their sequence similarity. Considering that in sugarcane and maize, *p*CA content is mainly associated with lignin, especially in the stems (Ralph, 2010; del Río et al., 2015), *ScAT1* expression pattern suggests a similar role to *BdAT1*, more likely related to FA-Ara levels than to *p*CA-Ara levels, although further investigations are required.

On the other hand, *ScAT5* low and tissue-specific expression levels (Supplementary Figures 5, 6) are consistent with this gene likely encoding a feruloyl-CoA monolignol transferase that participates in FA incorporation into lignin as suggested for its rice ortholog (*OsAT5*), because monolignol ferulates were detected in low levels in some grass species (Karlen et al., 2016). These results open the possibility of further studies on functional characterization of the identified genes and ultimately their use to achieve cost-effective hydroxycinnamate production in biorefineries.

Considering that it has been shown that rice OsAT10 is involved with *p*CA levels in the cell wall (Bartley et al., 2013), we sought to investigate whether there is a conserved role of AT10 in other grasses and the potential of using sugarcane *ScAT10* to generate C4 plants for biorefining purposes, with the goal of producing both bioenergy and *p*CA as a high-value co-product. The ScAT10 protein sequence similarity to that from other grasses (Supplementary Figure 7 and Supplementary Table 4A) and the general conserved expression pattern of *AT10* orthologous genes observed among sugarcane, maize, and rice (Supplementary Figure 8) supported the contention that AT10 enzymes likely have a similar role, at least in these grasses.

We have shown that *ScAT10* has a similar function to rice *OsAT10* (Bartley et al., 2013), associated with the content of both *p*CA and FA specifically linked to GAX. The effect observed for the overexpression of *ScAT10* in senescent tissues of maize (up to 75% *p*CA increase in senescent culms) (Figure 2) was similar to that observed for *OsAT10* overexpression in rice straw (80% *p*CA increase) (Bartley et al., 2013). However, it contrasted with that observed for the overexpression of *OsAT10* in senescent leaves of switchgrass, in which *p*CA released had a slight decrease of 15%, whereas the less lignified green leaves showed an increase in *p*CA (∼30%) (Li et al., 2018). Nonetheless, as *p*CA linked to GAX or lignin fractions was not distinguished in the switchgrass study (Li et al., 2018), it is possible that lignin *p*CA, not GAX, could have specifically decreased (possibly due to changes in the phenylpropanoid pathway), resulting in an overall slight decrease in the *p*CA levels of the more lignified senescent leaf.

Our data on neutral sugars measured in cell walls of transgenic lines indicate the absence of any compensatory effect in glucose and GAX sugar contents of the maize culms (Figure 5), similar to findings for the overexpression of *OsAT10* in switchgrass (Li et al., 2018). In contrast, *OsAT10* overexpression in rice resulted in a 20% increase in cell wall glucose (Bartley et al., 2013), suggesting that the effect observed in rice was specific for this species.

As previously discussed (Bartley et al., 2013), the observed decrease in FA could be a result of the competition of *p*CA and FA for arabinose, so that an increase in *p*CA-Ara resulted in fewer arabinose sites available for FA linkage. It is also possible that an increase in *p*CA incorporation into the cell wall could result in less feruloyl-CoA available for xylan modification, as *p*CA is a precursor in the phenylpropanoid pathway that leads to FA biosynthesis. Nevertheless, as for OsAT10, a characterization of ScAT10 enzyme activity is still needed. It is unknown if the *p*CA is transferred to monomeric arabinose (or an activated form, such as UDP-Ara) or directly into the arabinoxylan polymer. As most BAHD acyltransferases are cytoplasmatic and xylan synthesis occurs in the Golgi, ScAT10 could be transferring *p*CA to a cytoplasmatic precursor (Buanafina, 2009; Li et al., 2018).

Although there was a decrease in FA, we did not find significant increase in biomass digestibility of transgenic lines (Supplementary Figure 10), without using pretreatment. This suggests that even with less FA-Ara, other characteristics, such as high lignification of secondary cell walls, restrain direct polysaccharide hydrolysis of the samples. Pretreatment is required to release significant amounts of hemicellulose and/or lignin to allow proper enzymes access to cellulose (Himmel et al., 2007).

The impact of overexpressing *ScAT10* in the *p*CA and FA contents can be properly explored in biorefineries producing both bioenergy and *p*CA as a high-value product. Biofuels could be produced by fermentation of the cell wall sugars. Although the digestibility of non-pretreated cell walls was not enhanced in the maize transgenic lines (Supplementary Figure 10), there is great potential for further evaluation of *AT10* overexpressed plants under selected pretreatments. Decreased FA content can result in less cell wall cross-linked components, which could allow using milder than usual pretreatment conditions. Some studies have already described beneficial effects of genetically engineered lines with decreased FA contents associated with biological (Bartley et al., 2013) and chemical pretreatments (de Souza et al., 2019).

In the biorefinery context, *p*CA could be recovered from the pretreatment liquors (Timokhin et al., 2020). However, a techno-economic analysis showed that the production of hydroxycinnamic acids from biomass processed in alkaline conditions is currently not viable as purification yields are too low with current methods (Karlen et al., 2020). To achieve cost-effective hydroxycinnamate production, the authors suggested genetic engineering of the feedstock to produce high concentrations of a single type phenolic acid. Expression levels of several BAHD genes could be altered in order to manipulate the hydroxycinnamate content in biomass, and the best strategy would probably involve more than one gene in conjunction. Among BAHD genes, *ScAT10* is one of the best candidates to that end, as its overexpression is related not only to increased *p*CA but also to a significant decrease in FA levels. Our results indicate that for maize lines overexpressing *ScAT10*, from the total mean content of *p*CA and FA in the cell wall, 93% corresponds to *p*CA, against 79% in control lines. This suggests high *p*CA/FA ratios in pretreatment liquors of autohydrolysis, acid catalysis, and alkaline processes (van der Pol et al., 2015), which opens the possibility for *p*CA recovery at high yields and purity. Therefore, we have generated a C4 plant that is promising for the achievement of cost-effective production of biofuels and *p*CA in a biorefinery.

## Data Availability Statement

The original contributions presented in the study are included in the article/Supplementary Material, further inquiries can be directed to the corresponding author/s.

## Author Contributions

AFa designed and performed the experiments, analyzed the data, and wrote the manuscript. DR designed and contributed with the destination vectors and assisted in the cloning process. MS performed the digestibility analysis and assisted in the experiments. SK and JR contributed with mild acid hydrolysis and DFRC experiments. DR-P provided the sugarcane genomics data. RV provided the sugarcane transcriptomic and its expression data. TS helped in the experiments, data analysis, and interpretation and revised the manuscript. AFe provided the samples, conceived the study, and revised the manuscript. RH provided the experimental tools, performed the digestibility analysis, and helped in the data analysis and interpretation. ER designed, conceived the strategy, directed, and supervised this study, helped in the experiments, and wrote and revised the manuscript. All authors read and approved the final manuscript.

## Disclaimer

Mention of trade names or commercial products in this article is solely for the purpose of providing specific information and does not imply recommendation or endorsement by the United States Department of Agriculture.

## Conflict of Interest

DR was employed by the company Lytic Solutions, LCC, Madison, WI, United States. The remaining authors declare that the research was conducted in the absence of any commercial or financial relationships that could be construed as a potential conflict of interest.

## Footnotes

1 https://web.expasy.org/translate/

2 https://phytozome.jgi.doe.gov/pz/portal.html

3 https://itol.embl.de/

4 http://bar.utoronto.ca

5 http://www.biotech.iastate.edu/ptf/

6 https://www.r-project.org/
