# Supplementary material for: Overexpression of a Sugarcane BAHD Acyltransferase Alters Hydroxycinnamate Content in Maize Cell Wall
Source: Front Plant Sci. 2021 Apr 21;12:626168. doi: 10.3389/fpls.2021.626168 (PMC8117936; doi:10.3389/fpls.2021.626168)
Supplement: Supplementary file 1 [file Data_Sheet_1.docx]

Supplementary Material

# Supplementary Figures and Tables

## Supplementary Figures


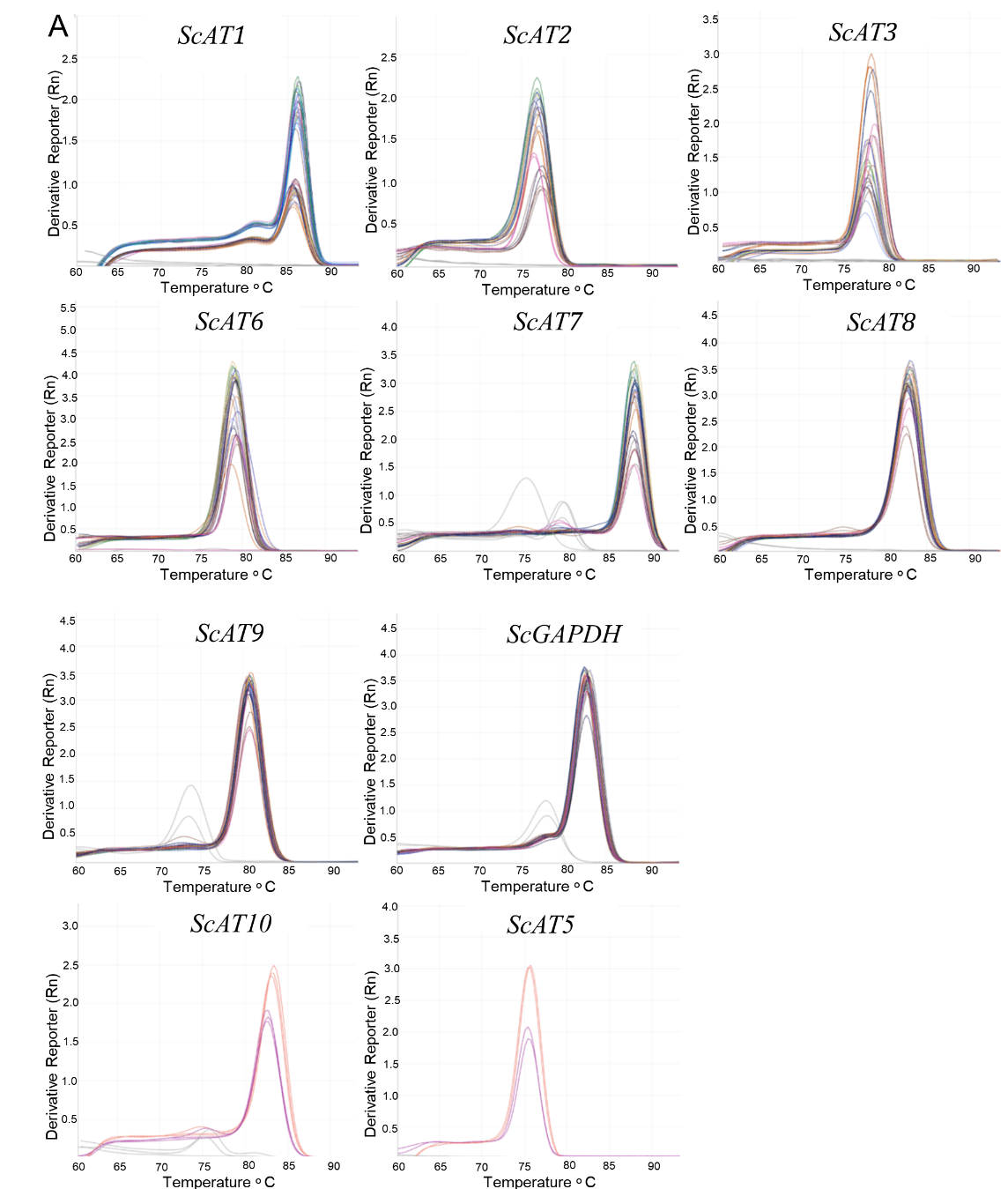


**Supplementary Figure 1A.** Melting curve analysis showing specificity of primers used on RT-qPCR reactions. A - Analysis of each identified sugarcane Mitchell clade A gene and reference gene *ScGAPDH*. All *ScAT1* to *ScAT9* and *ScGAPDH* genes from sugarcane were analyzed in young internode, while *ScAT10* and *ScAT5* genes were analyzed in immature leaf tissues. Plots show Derivative Reporter (Rn) versus Temperature (°C), for each primer pair. Colored peaks represent samples and curves in beige represent negative control.


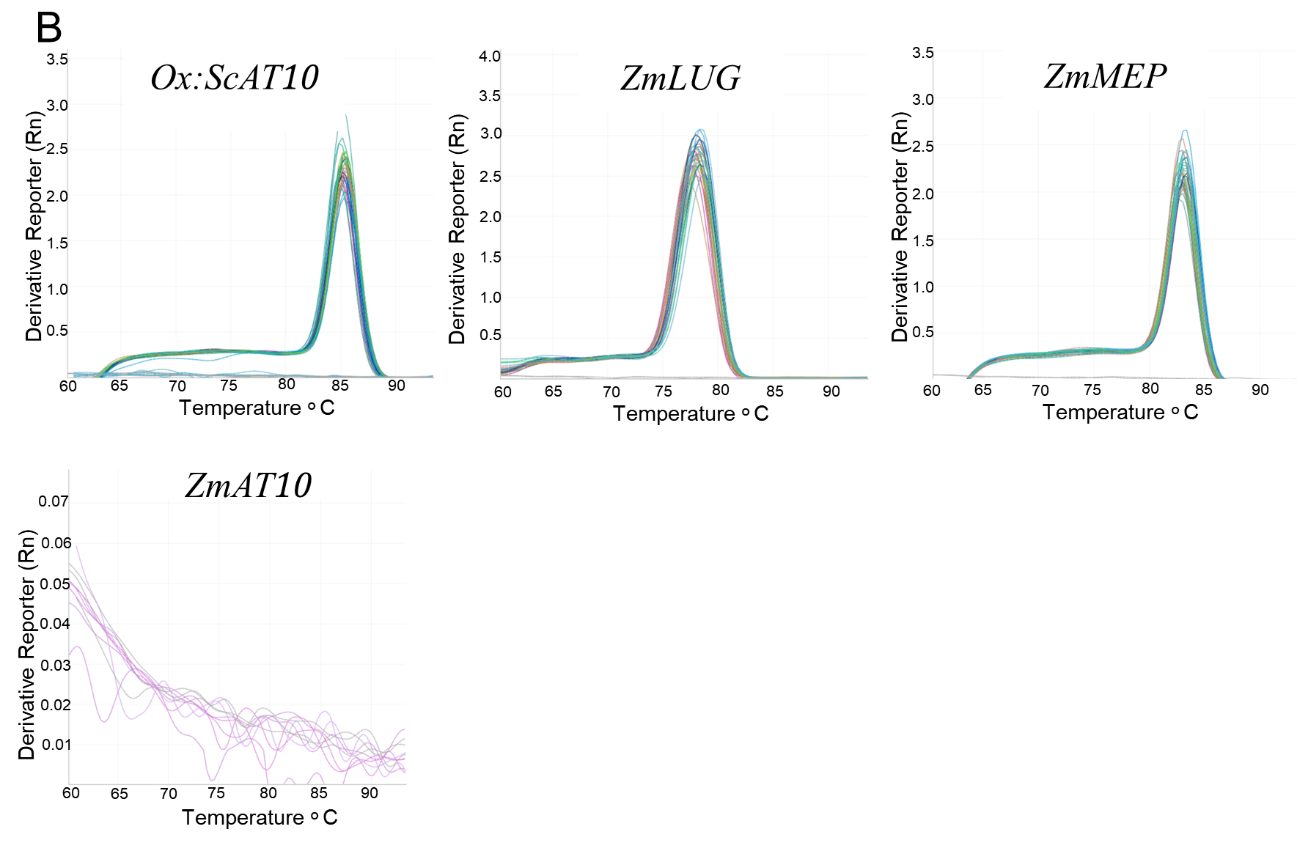


**Supplementary Figure 1B.** Melting curve analysis showing specificity of primers used on RT-qPCR reactions. B - Melting curve analysis of sugarcane transgene *ScAT10* and maize endogenous *ZmAT10* and reference genes *ZmLUG* and *ZmMEP* in RT-qPCR analysis of transgenic maize lines (*Ubi:ScAT10*). Plots show Derivative Reporter (Rn) versus Temperature (°C), for each primer pair. Colored peaks represent samples and curves in beige represent negative control.


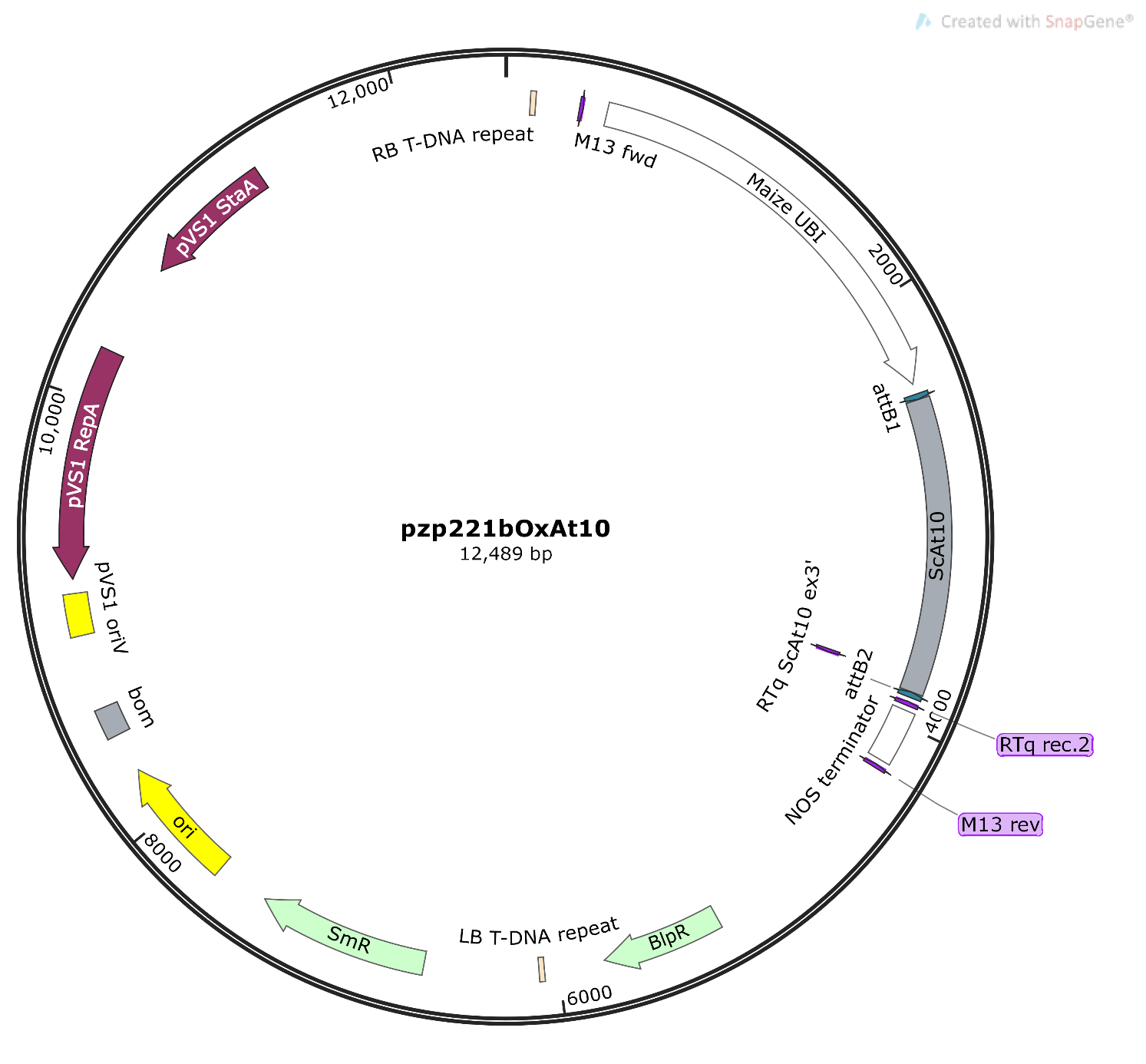


**Supplementary Figure 2.** *Pzp221b:Ox:ScAT10* (*ScAT10* overexpression) construction map. pCS1 StaA/ RepA, stability, and replication protein from *Pseudomonas* plasmid pVS1. Bom, basis of mobility region of pBR322. RB T-DNA, Right border repeat from T-DNA. Maize UBI, maize ubiquitin 1 promoter. *attb1/attb2*, Gateway^®^ recombination sites. *ScAT10*, sugarcane *AT10* cDNA. RTq rec2/RTq ScAT10 ex3`, primers used in RT-qPCR analysis for expression levels determination. NOS terminator, nopaline synthase transcription terminator. BlpR, Bialaphos resistance gene. LB T-DNA, Left border repeat from T-DNA. SmR, Spectinomycin and Streptomycin resistance gene (*aadA*). Number represent the length in base pairs.


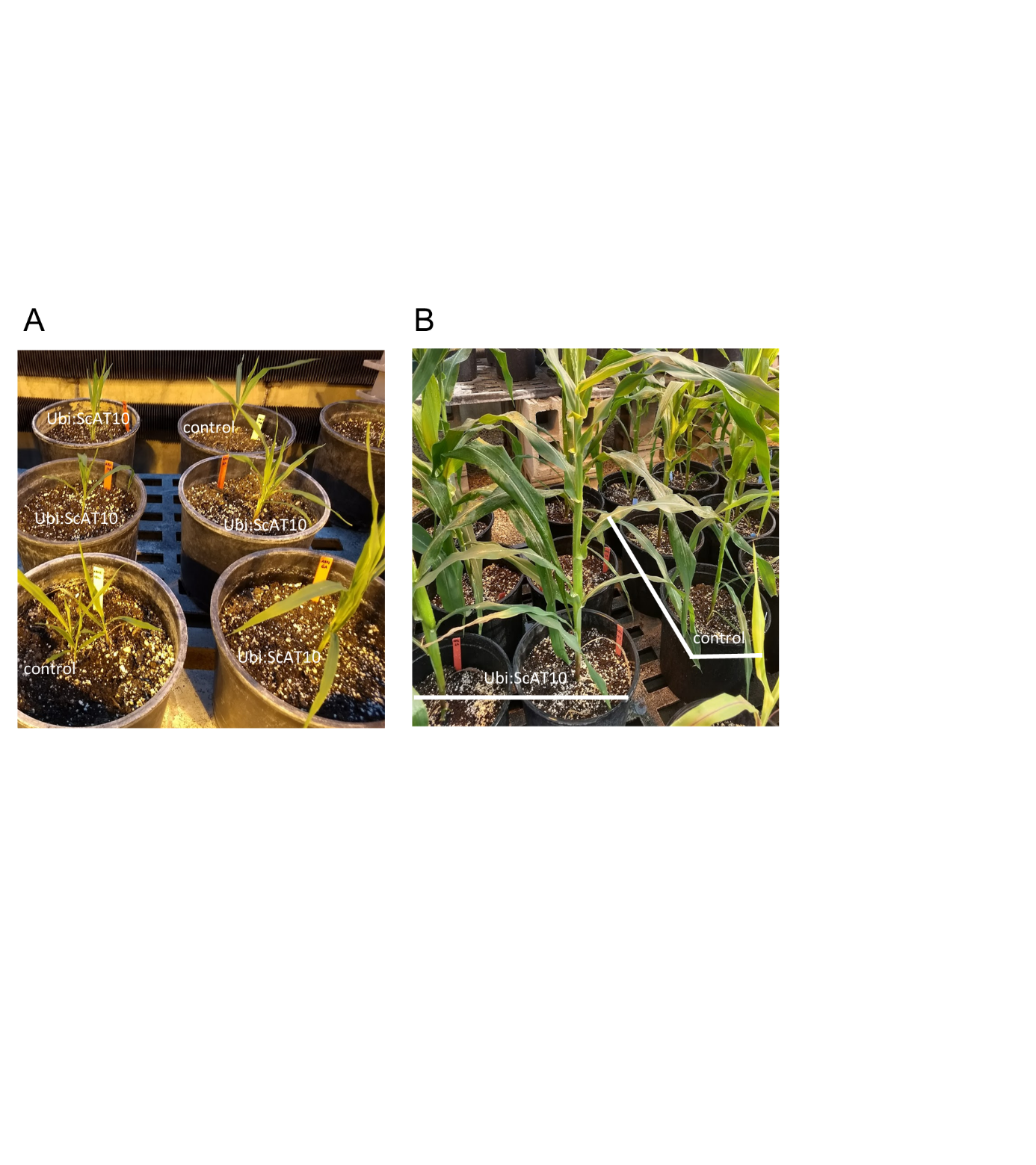


**Supplementary Figure 3.** Growth of maize transgenic and control lines. A - Plants after 1-month growth, cultivated in 11.5 L pots, in the greenhouse (16 h light, 8 h dark regime). B - Adult plants after 2-month growth.


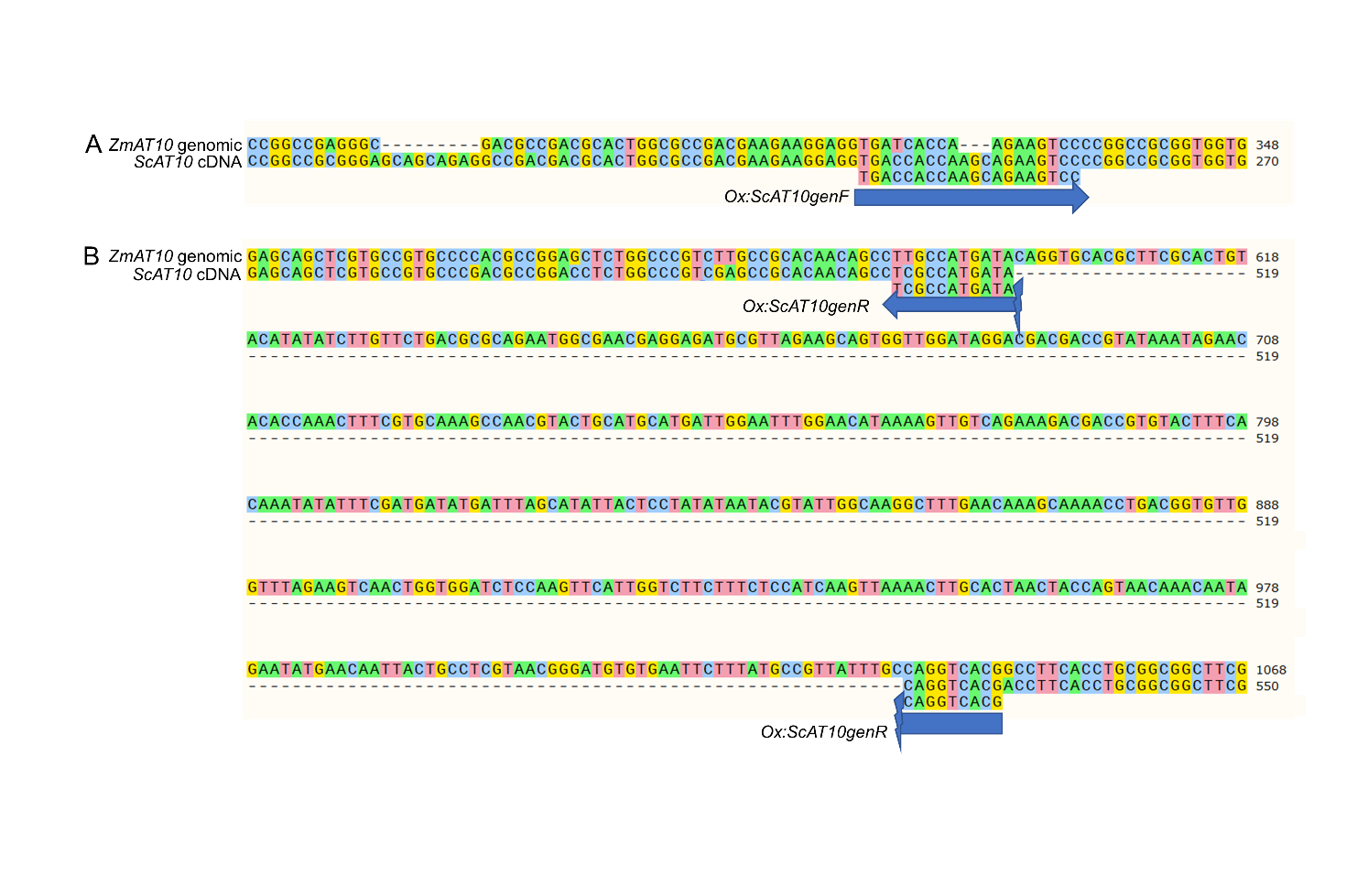


**Supplementary Figure 4.** Alignment of endogenous maize *ZmAT10* gene and the *ScAT10* cDNA region used to design primers *Ox:ScAT10genF/R* to genotype transgenic lines, showing primer specificity. A - Alignment showing *Ox:ScAT10genF* primer binding region. B - Alignment showing *Ox:ScAT10genR* primer binding region. The highlight in orange represents a break in the primer sequence, showing that the primer does not anneal in the genomic intron region (in brackets).


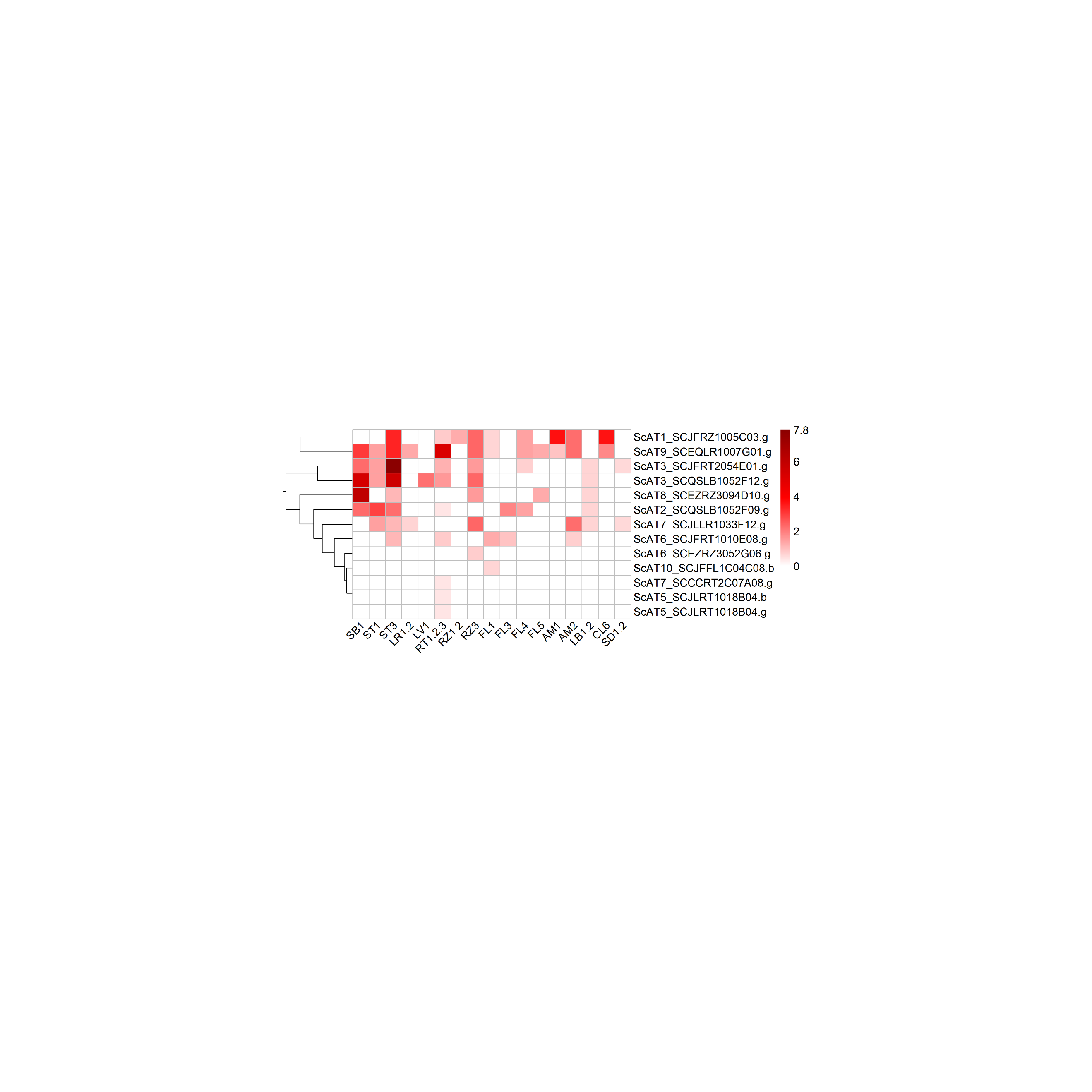


**Supplementary Figure 5.** Expression profile of sugarcane Mitchell clade A genes. Heatmap data of normalized reads for distinct EST libraries from SUCEST. Each library corresponds to a distinct tissue: SB1 Stalk bark upper; ST1 First apical internode; ST3 Fourth apical internode; LR1-2 Leaf-roll; LV1 Etiolated leaves; RT 1-2-3 Root; RZ1-2 Shoot root trans-zone; RZ3 Shoot-root trans-zone; FL1 Inflorescence at beginning; FL3 Base of developing inflorescence; FL4 Developed inflorescence; FL5 Developed inflorescence; AM1 Apical meristem (mature); AM2 Apical meristem (immature); LB1-2 Lateral bud; CL6 Cauli-temperature stress; SD1-2 Developing seeds.


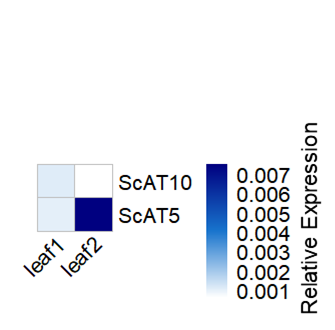


**Supplementary Figure 6.** RT-qPCR relative expression levels of *ScAT10* and *ScAT5* in immature leaf tissues of sugarcane hybrid H321. Values are relative expression normalized with reference gene *GAPDH* (2^-ΔCt).


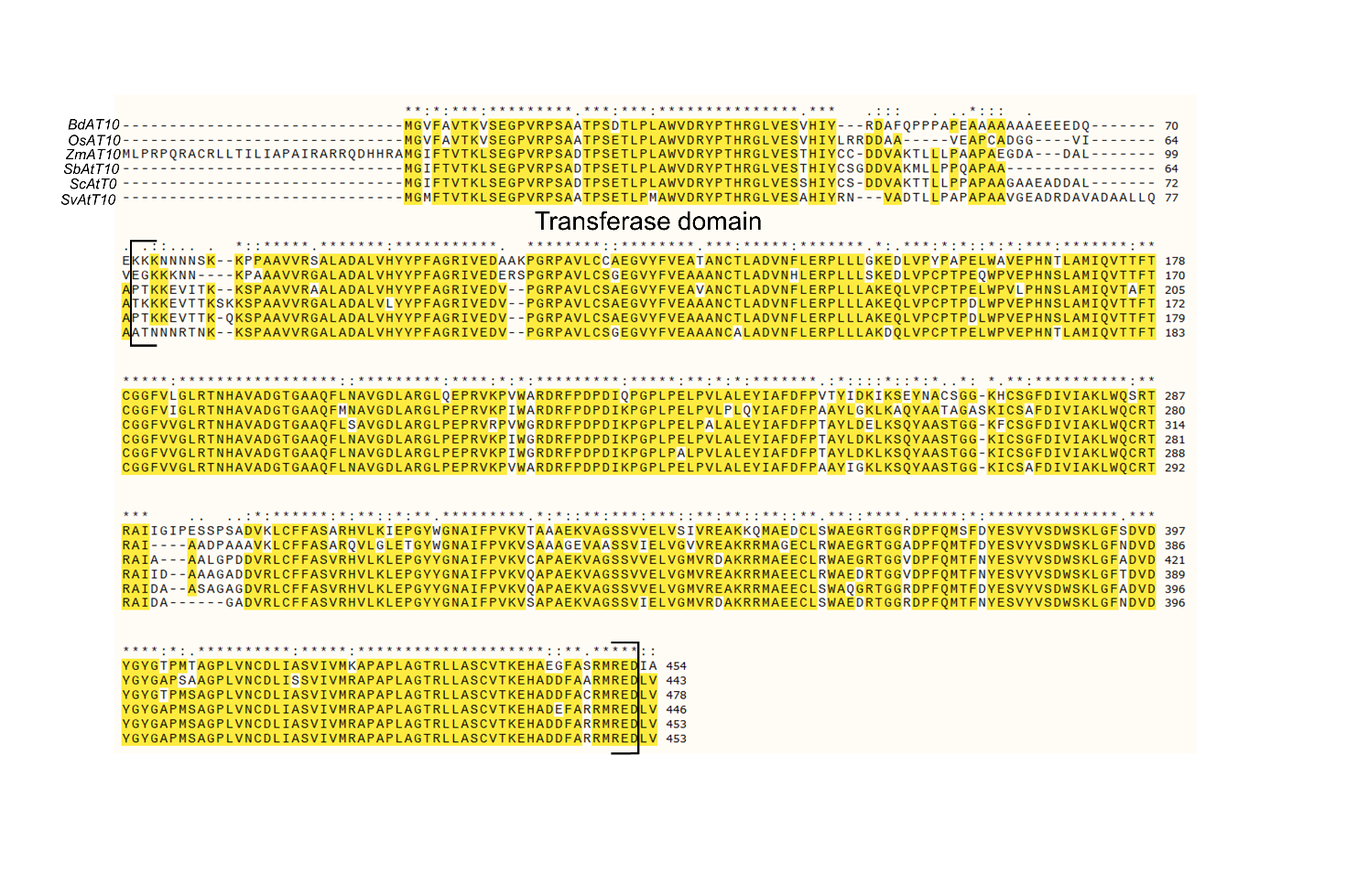


**Supplementary Figure 7.** Multiple sequence alignment of AT10 sequences from sugarcane and other grasses. Bd, Os, Sb, Sc, Sv, and Zm is abbreviation from Brachypodium, rice, sorghum, sugarcane, Setaria, and maize. The region containing PFAM transferase domain of AT10 sequences is indicated by brackets.

**
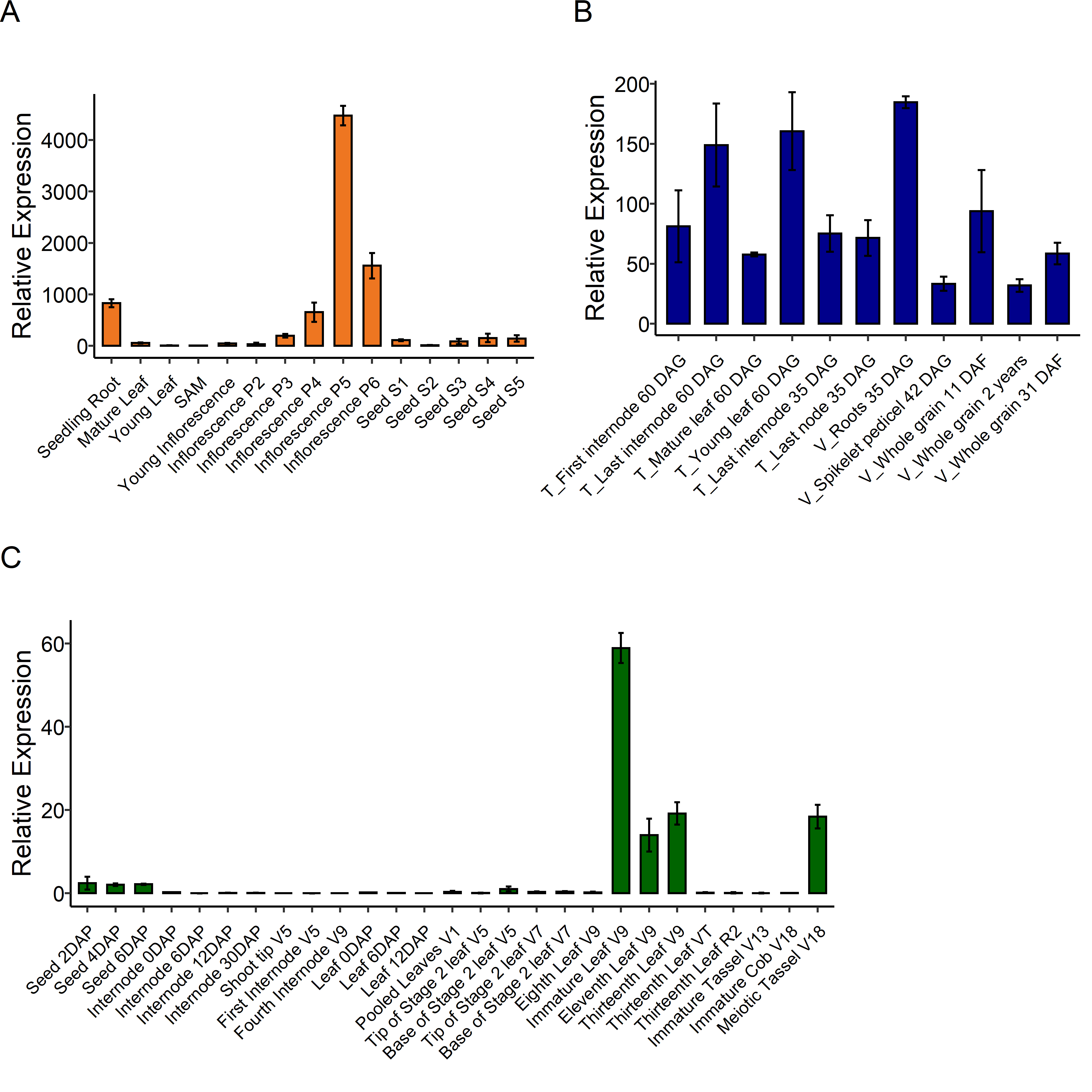
**

**Supplementary Figure 8.** *In silico* expression profile of *AT10* ortholog genes in maize, rice, and Brachypodium. (**A**) Micro-array expression data of *OsAT10* in distinct tissues from rice (Jain et al., 2007). (**B**) Micro-array expression data of *BdAT10* in distinct tissues from Brachypodium (Winter et al., 2007; Sibout et al., 2017). T- Plants cultivated in growth chamber in Toulouse, France. V- Plants grown in green house in Versailles, France. (**C**) RNA-seq expression data of *ZmAT10* in distinct tissues from maize B73 (Hoopes et al., 2019). Analysis obtained from Bar Utoronto eFP Browser tool (http://bar.utoronto.ca).


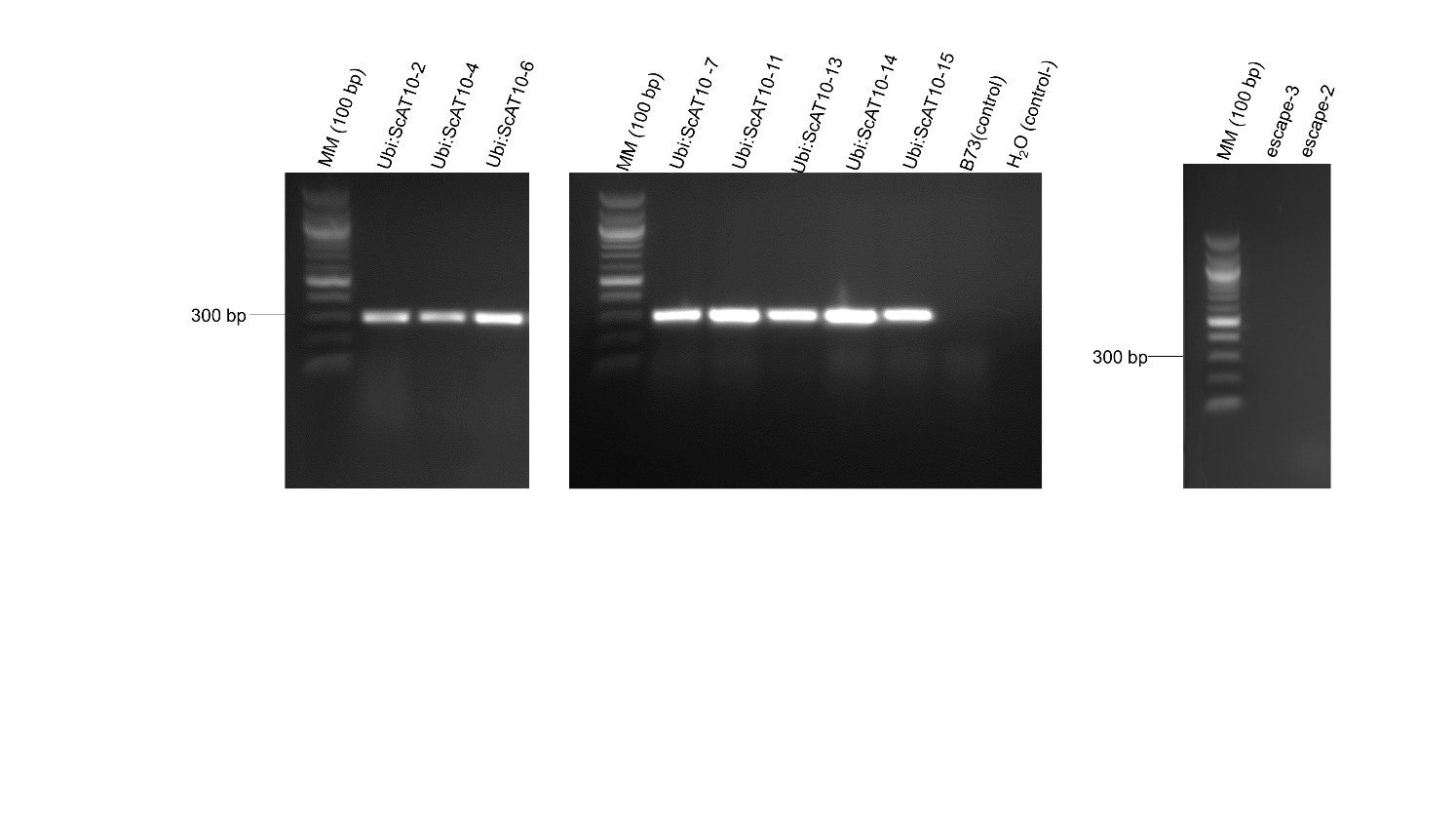


**Supplementary Figure 9.** PCR genotyping of transgenic maize lines. *Ubi:ScAT10* lines are positive for the presence of the *ScAT10* insert, whereas *escape-2* and *escape-3* are negative (empty vectors). MM - Molecular weight marker, 100 bp. Electrophoresis was performed using a 3% agarose gel stained with ethidium bromide.


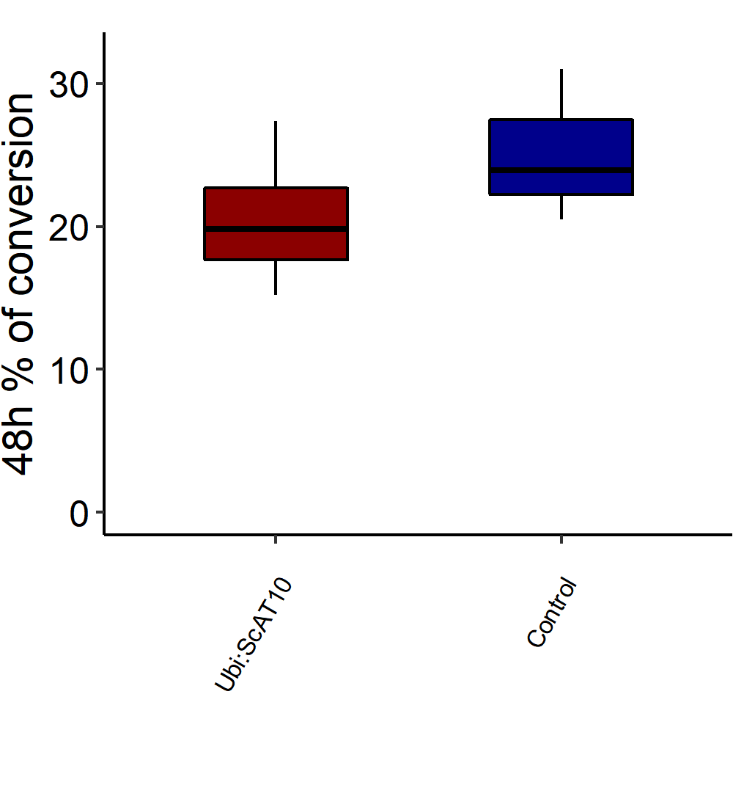


**Supplementary Figure 10.** Digestibility of cell walls from transgenic *Ubi:ScAT10* and control lines treated with cellulase and xylanase mix. Values represent average percent of cell wall sugar conversion after 48 h hydrolysis. Red boxes represent the distribution of values for transgenic lines (*Ubi:ScAT10-2,-6,-11,* and *-14,* n=4 plants). Blue boxes represent the distribution of values for control lines (empty vector *escape-2* and *escape-3*, and wild-type (WT) B73, n=3 plants). Each box delimits values within the first and third quartile range. The horizontal black lines in the boxes represent median values. Whiskers represent the data range. Means of transgenic lines were not significantly different from mean of controls (empty and B73) at *p*<0.05 (Welch’s t-test).

## Supplementary Tables

**Supplementary Table 1.** DNA primers for cloning, genotyping, and RT-qPCR analyses.

| **Name** | **Sequence** | **Purpose** |
| --- | --- | --- |
| ScAT1 F | TTCCCTCCGTTGTGTACCTG | RT-qPCR sugarcane |
| ScAT1 R | TTCCCACTGTGGAATGGAAT |  |
| ScAT2 F | GCCAAGTTCGCGTAGATCAT | RT-qPCR sugarcane |
| ScAT2 R | CGTTTGCATCCAACAATGTC |  |
| ScAT3 F | GCATCATGACCATGTGCGTC | RT-qPCR sugarcane |
| ScAT3 R | CCAAACTTGCACCTGGATGC |  |
| ScAT5 F | CAGTGTGTGGAGGAGAAGCA | RT-qPCR sugarcane |
| ScAT5 R | CCAATGAGGCTACTGGTCAA |  |
| ScAT6 F | CAGGTCGGTCTGTGTTGGAA | RT-qPCR sugarcane |
| ScAT6 R | AACCCCACCCATCAGTTAGC |  |
| ScAT7 F | CAGGGGCCAGGCTCATCA | RT-qPCR sugarcane |
| ScAT7 R | ACCCATGAACAAACGGGAGG |  |
| ScAT8 F | GCCTTCCACAAGGACATGAT | RT-qPCR sugarcane |
| ScAT8 R | TCATGGGATACAGCAACGAA |  |
| ScAT9 F | GCGTCACCAAGGAGCACT | RT-qPCR sugarcane |
| ScAT9 R | CAGTTTCCCCGTTTCATCAT |  |
| ScAT10 F | GAGGACCTCGTCTGATACGATA | RT-qPCR sugarcane |
| ScAT10 R | TCAGCACACTCACACGTAGG |  |
| ScGAPDH F | TTGGTTTCCACTGACTTCGTT | RT-qPCR sugarcane  reference gene |
| ScGAPDH R | CTGTAGCCCCACTCGTTGT | GAPDH (SCCCCL3001G02.g) |
| AttB1-OxShAT10 F | GGGGACAAGTTTgtacaaaaaagcaggctTT  ATGGGCATCTTCACGG | Amplify *AttbScAT10* product for gateway cloning |
| AttB2-OxShAT10 R | GGGGACCACTTTGTACAAGaaagctgggtAT  CAGACGAGGTCC |  |
| *Ox:ScAT10gen F* | TGACCACCAAGCAGAAGTCC | Genotyping *Ox:ScAT10* (*Ubi:ScAT10* lines) |
| *Ox:ScAT10gen R* | CGTGACCTGTATCATGGCGA |  |
| *RTq ScAT10 ex3'.2* | AGCTGCGTCACCAAGGAG | RT-qPCR analysis of *ScAT10* |
| *RTq rec.2* | CTAGTAACGGCCGCCAGTG | expression |
| *RTq_LUG_F* | CAGGGAAGGTTGCCTCAGTA | RT-qPCR reference gene |
| *RTq_LUG_R* | GCGTCATGTGGTCATTTTTG | LEUNIG (GRMZM2G425377) |
| *RTq_MEP_F* | CCATCTGTCTGGGTCAGGAT | RT-qPCR reference gene |
| *RTq_MEP_R* | TTTGATGCTCCAGGCTTACC | Membrane  protein PB1A10.07c |
|  |  | (GRMZM2G018103) |

**Supplementary Table 2A.** Product size of each RT-qPCR amplicon and average primer pair efficiency as estimated by LinReg PCR for primers used in RT-qPCR expression analysis of Mitchell clade A genes in sugarcane samples.

| Name | Product size | Efficiency |
| --- | --- | --- |
| *ScAT1* | 148 bp | 1.88 |
| *ScAT2* | 140 bp | 1.85 |
| *ScAT3* | 143 bp | 1.92 |
| *ScAT5* | 141 bp | 1.83 |
| *ScAT6* | 127 bp | 1.92 |
| *ScAT7* | 124 bp | 1.90 |
| *ScAT8* | 118 bp | 1.79 |
| *ScAT9* | 109 bp | 1.92 |
| *ScAT10* | 105 bp | 1.86 |
| *ScGAPDH* | 122 bp | 1.91 |

**Supplementary Table 2B.** Product size of each RT-qPCR amplicon and average primer pair efficiency as estimated by LinReg PCR for primers used in RT-qPCR expression analysis of *Ubi:ScAT10* in maize transgenic and control lines.

| Name | Product size | Efficiency |
| --- | --- | --- |
| *RTq ScAT10 ex3'.2/ RTq rec.2* | 128 | 1.68 |
| *RTqZmLUG* | 110 | 1.90 |
| *RTq ZmMEP* | 129 | 1.74 |
| *RTq ZmCUL* | 125 | 1.80 |

**Supplementary Table 3A.** Sugarcane sequences from genomic sources identified in the Mitchell Clade A and alignment information with sorghum ortholog.

| Name | Sorghum | Sugarcane genomic | Source | Identity* | | Cover  ** | Length sugarcane (aa) | Length sorghum(aa) |
| --- | --- | --- | --- | --- | --- | --- | --- | --- |
| AT1 | Sobic.003G219800 | deg7180000190712_g2410^a^ | SP8032-80 | 97 | 100 | | 429 | 426 |
| AT2 | Sobic.003G219700 | scf7180000349327_g7029^a^ | SP8032-80 | 95 | 99 | | 425 | 428 |
|  |  | scf7180000356993_g1930 | SP8032-80 | 98 | 37 | | 162 |  |
|  |  | scf7180000354309_g6422 | SP8032-80 | 99 | 37 | | 162 |  |
| AT3 | Sobic.009G034300 | Sh09_p003100^a^ | R570 STP | 92 | 99 | | 427 | 436 |
|  |  | singlet_100000184749_g762 | SP8032-80 | 89 | 59 | | 152 |  |
|  |  | Sh_202B14_p000080 | R570 BAC | 90 | 55 | | 236 |  |
|  |  | Sh_202B14_p000070 | R570 BAC | 93 | 69 | | 294 |  |
| AT5 | Sobic.008G068300 | scf7180000355999_g504^a^ | SP8032-80 | 94 | 64 | | 287 | 440 |
| AT6 | Sobic.003G043600 | Sh03_p003210^a^ | R570 STP | 95 | 100 | | 436 | 444 |
|  |  | Sh_219N24_p000050 | R570 BAC | 90 | 34 | | 202 |  |
| AT7 | Sobic.009G065600 | Not found | - | - | - | | - | 441 |
| AT8 | Sobic.010G180100 | Sh10_p012420^a^ | R570 | 94 | 100 | | 441 | 438 |
|  |  | Sh_217G03_p000010 | R570 BAC | 91 | 36 | | 189 |  |
|  |  | Sh_224K09_p000080 | R570 BAC | 93 | 100 | | 441 |  |
| AT9 | Sobic.003G037800 | deg7180000278811_g7338^a^ | SP8032-80 | 98 | 100 | | 417 | 417 |
|  |  | scf7180000354540_g6769 | SP8032-80 | 98 | 63 | | 265 |  |
|  |  | deg7180000229535_g7252 | SP8032-80 | 98 | 63 | | 265 |  |
| AT10 | Sobic.010G179900 | scf7180000358018_g3307^a^ | SP8032-80 | 92 | 100 | | 453 | 446 |

References for each sugarcane sequence source: SP803280 - Riano-Pachón and Matiello, 2017, R570 - Garsmeur *et al.*, 2018. Alignment performed using BLASTp with sorghum as query and sugarcane as subject. * Identity is the percentage of identities in the aligned region by BLASTp. ** Cover is the percent of sorghum sequence covered by the alignment. ^a^ indicates code containing sequence used for ML tree in Fig. 1A.

**Supplementary Table 3B.** Sugarcane sequences from transcriptomic sources identified in the Mitchell Clade A and alignment information with sorghum ortholog.

| Name | sorghum | Sugarcane genomic | Source | Identity* | Cover  ** | Length sugarcane (aa) | Length sorghum  (aa) |
| --- | --- | --- | --- | --- | --- | --- | --- |
| AT1 | Sobic.003G219800 | comp192319_c0_seq1 | leaf | 97 | 100 | 429 | 426 |
|  |  | Locus824.1_Confidence_1.000_Length_1566 | internode | 97 | 100 | 429 |  |
|  |  | SCJFRZ1005C03.g | SUCEST | 97 | 100 | 429 |  |
| AT2 | Sobic.003G219700 | comp196893_c0_seq1 | leaf | 96 | 99 | 424 | 428 |
|  |  | Locus3452.1_Confidence_1.000_Length_1064 | internode | 95 | 67 | 291 |  |
|  |  | Locus18879.1_Confidence_1.000_Length_637 | internode | 99 | 33 | 145 |  |
|  |  | SCQSLB1052F09.g | SUCEST | 95 | 99 | 425 |  |
| AT3 | Sobic.009G034300 | SCJFRT2054E01.g | SUCEST | 93 | 96 | 414 | 436 |
|  |  | SCQSLB1052F12.g | SUCEST | 87 | 65 | 212 |  |
|  |  | comp193913_c2_seq1 | leaf | 95 | 70 | 304 |  |
|  |  | comp193913_c3_seq1 | leaf | 86 | 41 | 135 |  |
|  |  | Locus323.15_Confidence_0.243_Length_1901 | internode | 92 | 99 | 432 |  |
| AT5 | Sobic.008G068300 | Locus5470.1_Confidence_1.000_Length_1343 | internode | 94 | 85 | 373 | 440 |
|  |  | SCJLRT1018B04.b | SUCEST | 89 | 32 | 142 |  |
|  |  | SCJLRT1018B04.g | SUCEST | 90 | 43 | 192 |  |
| AT6 | Sobic.003G043600 | SCJFRT1010E08.g | SUCEST | 99 | 51 | 250 | 444 |
|  |  | SCEZRZ3052G06.g | SUCEST | 84 | 36 | 162 |  |
| AT7 | Sobic.009G065600 | comp105233_c0_seq1 | leaf | 97 | 21 | 131 | 441 |
|  |  | Locus9463.10_Confidence_0.585_Length_1498 | internode | 88 | 91 | 395 |  |
|  |  | SCJLLR1033F12.g^a^ | SUCEST | 91 | 100 | 438 |  |
|  |  | SCCCRT2C07A08.g | SUCEST | 95 | 29 | 134 |  |
| AT8 | Sobic.010G180100 | comp203803_c0_seq1 | leaf | 66 | 80 | 308 | 438 |
|  |  | Locus6993.4_Confidence_0.826_Length_1710 | internode | 94 | 66 | 308 |  |
|  |  | SCEZRZ3094D10.g | SUCEST | 93 | 62 | 478 |  |
| AT9 | Sobic.003G037800 | comp186435_c0_seq1 | leaf | 98 | 100 | 417 | 417 |
|  |  | Locus3963.2_Confidence_1.000_Length_1583 | internode | 98 | 100 | 417 |  |
|  |  | SCEQLR1007G01.g | SUCEST | 98 | 100 | 417 |  |
| AT10 | Sobic.010G179900 | Locus7917.2_Confidence_1.000_Length_1272 | internode | 95 | 86 | 383 | 446 |
|  |  | SCJFFL1C04C08.b | SUCEST | 96 | 51 | 199 |  |

References for each sugarcane sequence source: SUCEST - Vettore *et al.*, 2003 (3); Vicentini *et al.*, 2012 (4), leaf - Cardoso-Silva *et al.*, 2014 (6), internode - Vicentini *et al.*, 2015 (5). Alignment performed using BLASTp with sorghum as query and sugarcane as subject. * Identity is the percentage of identities in the aligned region by BLASTp. ** Cover is the percent of sorghum sequence covered by the alignment. ^a^ indicates code containing sequence used for ML tree in Fig. 1A.

**Supplementary Table 4A.** Identity matrix for AT10 full-length amino acid sequences from Brachypodium, rice, maize, sorghum, sugarcane, and Setaria.

|  | Brachypodium | Rice | Maize | Sorghum | Sugarcane | Setaria |
| --- | --- | --- | --- | --- | --- | --- |
| Brachypodium | ID | 77.0% | 72.8% | 77.9% | 79.4% | 77.1% |
| Rice | 77.0% | ID | 74.2% | 81.0% | 80.5% | 79.2% |
| Maize | 72.8% | 74.2% | ID | 85.4% | 86.1% | 79.9% |
| Sorghum | 77.9% | 81.0% | 85.4% | ID | 92.9% | 85.9% |
| Sugarcane | 79.4% | 80.5% | 86.1% | 92.9% | ID | 87.3% |
| Setaria | 77.1% | 79.2% | 79.9% | 85.9% | 87.3% | ID |

Amino acid sequences from Brachypodium (Bradi13g36990), rice (LOC_Os06g39390), maize (GRMZM2G107027), sorghum (Sobic.010G179900), sugarcane (scf7180000358018_g3307), and Setaria (Sevir.4G238000). Alignment was performed using ClustalW and the identity matrix was created using BioEdit^®^.

**Supplementary Table 4B.** Identity matrix for AT10 transferase domain region amino acid sequences from Brachypodium, rice, maize, sorghum, sugarcane, and Setaria.

|  | Brachypodium | Rice | Maize | Sorghum | Sugarcane | Setaria |
| --- | --- | --- | --- | --- | --- | --- |
| Brachypodium | ID | 78.2% | 79.0% | 81.2% | 81.1% | 80.8% |
| Rice | 78.2% | ID | 79.3% | 83.0% | 82.5% | 82.8% |
| Maize | 79.0% | 79.3% | ID | 91.0% | 91.0% | 86.9% |
| Sorghum | 81.2% | 83.0% | 91.0% | ID | 94.7% | 89.2% |
| Sugarcane | 81.1% | 82.5% | 91.0% | 94.7% | ID | 90.0% |
| Setaria | 80.8% | 82.8% | 86.9% | 89.2% | 90.0% | ID |

Aminoacid sequences from Brachypodium (Bradi13g36990), Rice (LOC_Os06g39390), Maize (GRMZM2G107027), Sorghum (Sobic.010G179900), Sugarcane (scf7180000358018_g3307) and Setaria (Sevir.4G238000). Alignment was performed using ClustalW and the identity matrix was created using BioEdit^®^.

**Supplementary Table 5.** *p*-Coumaric acid (*p*CA), ferulic acid (FA) and ratio of *p*CA/FA content released by mild alkaline treatment, from culms of transgenic lines *Ubi:ScAT10* and control lines(empty vector *escape-2* and *escape-3*, and wild-type B73).

| Lines | *p*CA | FA | *p*CA/FA |
| --- | --- | --- | --- |
| *Ubi:ScAT10-2* | 29.0 ± 2.5 | 1.8 ± 0.2 *** | 15.9 ± 0.1 *** |
| *Ubi:ScAT10-4* | 32.9 ± 2.3 ** | 3.1 ± 0.1 * | 10.6 ± 0.3 ** |
| *Ubi:ScAT10-6* | 29.3 ± 1.6 | 2.0 ± 0.2 *** | 15.1 ± 0.6 *** |
| *Ubi:ScAT10-7* | 29.8 ± 0.5 | 3.7 ± 0.2 | 8.1 ± 0.2 |
| *Ubi:ScAT10-11* | 40.6 ± 1.0 *** | 1.5 ± 0.1 *** | 27.7 ± 0.7 *** |
| *Ubi:ScAT10-13* | 21.2 ± 0.4 | 3.1 ± 0.1 * | 6.9 ± 0.1 |
| *Ubi:ScAT10-14* | 29.5 ± 1.3 | 0.7 ± 0.1 *** | 43.2 ± 2.2 *** |
| *Ubi:ScAT10-15* | 20.8 ± 1.5 | 2.7 ± 0.2 ** | 7.7 ± 0.1 |
| *escape-2* | 27.8 ± 0.4 | 3.3 ± 0.3 | 8.6 ± 0.5 |
| *escape-3* | 18.6 ± 0.0 | 6.6 ± 0.2 | 2.8 ± 0.1 |
| B73-1 | 20.3 ± 0.2 | 6.8 ± 0.0 | 3.0 ± 0.0 |
| B73-2 | 26.0 ± 2.3 | 7.1 ± 0.5 | 3.6 ± 0.1 |
| B73-3 | 21.3 ± 0.1 | 6.3 ± 0.0 | 3.4 ± 0.0 |
| B73-4 | 25.9 ± 2.4 | 6.3 ± 1.0 | 4.1 ± 0.3 |

Values are g/Kg cell wall and represent means ± SEM (n=2 technical replicates). For each *Ubi:ScAT10* line, values followed by *, **, *** were significantly different from the mean of control lines at *p* <0.05, *p*<0.01 and *p*<0.001, respectively (ANOVA followed by post-hoc Dunnet’s test against the mean values of B73 and empty vector lines).

**Supplementary Table 6.** *p*-Coumaric acid linked to arabinose (*p*CA-Ara) and ferulic acid linked to arabinose (FA) content released by mild acidolysis, from culms of transgenic lines *Ubi:ScAT10* and control lines(empty vector *escape-2* and *escape-3*, and wild-type B73).

| Lines | *p*CA-Ara | FA-Ara |
| --- | --- | --- |
| *Ubi:ScAT10-2* | 2.3 ± 0.1 *** | 1.4 ± 0.1 *** |
| *Ubi:ScAT10-6* | 5.1 ± 0.2 *** | 1.2 ± 0.0 *** |
| *Ubi:ScAT10-11* | 4.9 ± 0.1 *** | 0.9 ± 0.0 *** |
| *Ubi:ScAT10-14* | 4.9 ± 0.1 *** | 0.4 ± 0.0 *** |
| *escape-2* | 0.1 ± 0.0 | 2.8 ± 0.2 |
| *escape-3* | 0.0 ± 0.0 | 4.7 ± 0.1 |
| B73 | 0.0 ± 0.0 | 3.6 ± 0.1 |

Values are g/Kg cell wall and represent means ± SEM (n=3 technical replicates). For each *Ubi:ScAT10* line, values followed by *** were significantly different from the mean of control lines at *p*<0.001 (ANOVA followed by post-hoc Dunnet’s test against the mean values of B73 and empty vector lines).

**Supplementary Table 7.** Non-acylated S, G, and H units, S-*p*CA and G-*p*CA amounts in lignin from culms of transgenic lines *Ubi:ScAT10* and control lines(empty vector *escape-2* and *escape-3*, and wild-type B73), as determined by DFRC.

| Lines | S-*p*CA | S | G | G-*p*CA | H |
| --- | --- | --- | --- | --- | --- |
| *Ubi:ScAT10-2* | 15.6 ± 1.1 | 5.0 ± 0.1 | 10.3 ± 0.0 | 0.3 ± 0.0 | 0.4 ± 0.0 |
| *Ubi:ScAT10-6* | 14.2 ± 0.8 | 5.1 ± 0.1 | 9.9 ± 0.2 | 0.3 ± 0.0 | 0.3 ± 0.0 |
| *Ubi:ScAT10-11* | 13.6 ± 0.5 | 5.2 ± 0.1 | 11.6 ± 0.0 | 0.4 ± 0.0 | 0.3 ± 0.0 |
| *Ubi:ScAT10-14* | 12.1 ± 0.4 | 4.3 ± 0.0 | 9.5 ± 0.0 | 0.3 ± 0.0 | 0.3 ± 0.0 |
| *escape-2* | 18.0 ± 0.2 | 5.5 ± 0.2 | 10.2 ± 0.2 | 0.4 ± 0.1 | 0.3 ± 0.0 |
| *escape-3* | 12.9 ± 0.1 | 4.7 ± 0.0 | 7.7 ± 0.2 | 0.2 ± 0.0 | 0.2 ± 0.0 |
| B73 | 16.6 ± 1.6 | 7.2 ± 0.1 | 12.6 ± 0.1 | 0.3 ± 0.0 | 0.5 ± 0.0 |

Values are g/Kg cell wall and represent means ± SEM (n=2 technical replicates). Values for each *Ubi:ScAT10* line were not significantly different from the mean of control lines at *p*<0.05 (ANOVA followed by post-hoc Dunnett’s test against the mean values of B73 and empty vector lines).

**Supplementary Table 8.** Glucose, Xylose and Arabinose content from culms of transgenic lines *Ubi:ScAT10* and control lines (empty vector *escape-2* and *escape-3*, and wild-type B73).

| Lines | Glucose | Xylose | Arabinose |
| --- | --- | --- | --- |
| *Ubi:ScAT10-2* | 443.9 ± 2.6 | 207.8 ± 5.9 | 28.6 ± 6.6 |
| *Ubi:ScAT10-6* | 488.2 ± 8.0 | 251.4 ± 5.6 | 29.1 ± 0.8 |
| *Ubi:ScAT10-11* | 439.2 ± 51.7 | 225.0 ± 29.0 | 23.0 ± 0.6 |
| *Ubi:ScAT10-14* | 505.4 ± 3.6 | 224.6 ± 0.6 | 21.8 ± 0.1 |
| *escape-2* | 470.2 ± 12.6 | 237.5 ± 0.6 | 38.0 ± 0.9 |
| *escape-3* | 387.8 ± 73.2 | 204.0 ± 28.5 | 31.9 ± 1.5 |
| B73 | 476.2 ± 14.4 | 211.4 ± 13.6 | 25.2 ± 0.6 |

Values are g/Kg cell wall and represent means ± SEM (n=2 technical replicates). Values for each *Ubi:ScAT10* line were not significantly different from the mean of control lines at *p*<0.05 (ANOVA followed by post-hoc Dunnett’s test against the mean values of B73 and empty vector lines).

**Supplementary Table 9.** Cell wall sugar conversion after 48h hydrolysis from transgenic *Ubi:ScAT10* and control lines (empty vector *escape-2* and *escape-3*, and wild-type B73) treated with cellulase and xylanase mix.

| Lines | % conversion |
| --- | --- |
| *Ubi:ScAT10-2* | 18.5 ± 4.8 |
| *Ubi:ScAT10-6* | 27.4 ± 5.6 |
| *Ubi:ScAT10-11* | 15.2 ± 4.1 |
| *Ubi:ScAT10-14* | 21.2 ± 2.4 |
| *escape-2* | 31.0 ± 2.8 |
| *escape-3* | 24.0 ± 3.4 |
| B73 | 20.5 ± 3.4 |

Values represent means ± SEM (n=2 technical replicates). Values for each line were not significantly different from the mean of controls at *p*<0.05 (ANOVA followed by post-hoc Dunnett’s test against the mean values of B73 and empty vector lines).
